# Tumor‐Targeting Cholesterol‐Decorated DNA Nanoflowers for Intracellular Ratiometric Aptasensing

**DOI:** 10.1002/adma.202007738

**Published:** 2021-02-08

**Authors:** Nayoung Kim, Eunjung Kim, Hyemin Kim, Michael R. Thomas, Adrian Najer, Molly M. Stevens

**Affiliations:** ^1^ Department of Materials Department of Bioengineering and Institute of Biomedical Engineering Imperial College London London SW7 2AZ UK; ^2^ Present address: Division of Bioengineering and Department of Bioengineering and Nano‐Bioengineering Incheon National University Incheon 22012 Republic of Korea; ^3^ Present address: London Centre for Nanotechnology and Department of Biochemical Engineering University College London London WC1H 0AH UK

**Keywords:** aptasensors, DNA flowers, Förster resonance energy transfer, ratiometric aptasensing, rolling circle amplification

## Abstract

Probing endogenous molecular profiles is of fundamental importance to understand cellular function and processes. Despite the promise of programmable nucleic‐acid‐based aptasensors across the breadth of biomolecular detection, target‐responsive aptasensors enabling intracellular detection are as of yet infrequently realized. Several challenges remain, including the difficulties in quantification/normalization of quencher‐based intensiometric signals, stability issues of the probe architecture, and complex sensor operations often necessitating extensive structural modeling. Here, the biomimetic crystallization‐empowered self‐assembly of a tumor‐targetable DNA–inorganic hybrid nanocomposite aptasensor is presented, which enables Förster resonance energy transfer (FRET)‐based quantitative interpretation of changes in the cellular target abundance. Leveraging the design programmability and high‐throughput fabrication of rolling circle amplification‐driven DNA nanoarchitecture, this designer platform offers a method to self‐assemble a robust nanosensor from a multifunctionality‐encoded template that includes a cell‐targeting aptamer, a ratiometric aptasensor, and a cholesterol‐decorating element. Taking prostate cancer cells and intracellular adenosine triphosphate molecules as a model system, a synergistic effect in the targeted delivery by cholesterol and aptamers, and the feasibility of quantitative intracellular aptasensing are demonstrated. It is envisioned that this approach provides a highly generalizable strategy across wide‐ranging target systems toward a biologically deliverable nanosensor that enables quantitative monitoring of the abundance of endogenous biomolecules.

## Graphical abstract

A programmable DNA–inorganic hybrid nanocomposite (DNA nanoflower) intracellular aptasensor with sequence‐encoded multifunctional features is realized by implementing enzymatic amplification‐driven biomimetic crystallization. This platform is capable of ratiometric and quantitative intracellular detection upon targeted delivery and holds substantial promise as strategically self‐assembled DNA nanosensors for interrogating cellular analytes whose changes in abundance are explained as a continuous function.

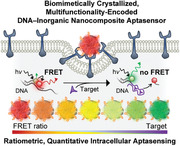

The advent of aptamers, nucleic acid analogs of antibodies, has drastically expanded the spectrum of bio/chemical targets that nucleic‐acid‐based probes can detect.^[^ ^1^ ^]^ Capitalizing on high affinity and target selectivity, DNA aptamers have been engineered into diverse designs of nanobiosensors, widely referred to as aptasensors.^[^ ^2^ ^]^ When it comes to functioning in intracellular environments, nanoparticle‐based fluorescence aptasensors have shown particular success, mainly attributed to their high cellular uptake and resistance to degradation due to a dense layer of highly oriented DNA strands. Moreover, the fluorescence‐based sensing in principle shows relatively higher sensitivity than other signaling strategies such as absorbance‐ and luminescence‐based signals.^[^ ^3^ ^]^ In a typical design, nanoparticles serve as a nanocarrier (often also as a quencher) where aptamers and signaling strands (where a fluorescent tag is usually conjugated) are either covalently or noncovalently functionalized.^[^ ^4^ ^]^ In the presence of target analytes, reporter fluorophores or molecular switches are separated, leading to switching on or off the fluorescence signals. Despite the progress made, a major challenge for such nanoparticle‐based strategies is that the switchable signals are typically intensiometric. This associates with fundamental difficulties in quantification/normalization of signals and interference with false‐positive signals arising from leaching signaling strands, cellular autofluorescence, or imaging artifacts. If there is a distinct difference in cell types and uptake efficiency/route of the delivered particles, intensiometric methods further limit an accurate comparison across different in vitro experimental conditions.^[^ ^3^ ^]^

Indeed, there has been an emerging move, yet still limited, toward ratiometric intracellular aptasensors by introducing a reference dye as an internal control^[^ ^5^ ^]^ or exploiting a donor/acceptor dye pair based on the Förster resonance energy transfer (FRET) mechanism.^[^ ^6^, ^7^, ^8^ ^]^ Particularly, FRET‐based DNA nanoassemblies constructed based on Watson–Crick base pairing, called DNA triangular prism (DTP) and tetrahedral DNA nanostructures (TDN), have emerged as potential candidates based on the precise modeling of a strand‐deforming operation. For instance, a DTP aptasensor for intracellular adenosine triphosphate (ATP) employed a split‐aptamer strategy, where two fragmented aptamer strands were labeled with a donor and acceptor dye, respectively, bringing the dyes in close proximity upon the target binding.^[^ ^6^ ^]^ Recent studies reported lysosomal ATP sensors based on the operation of the i‐motif DNA, a tetrameric DNA structure assembled by four intercalated DNA strands in the presence of protons.^[^ ^7^, ^8^ ^]^ Within the lysosomal acidic environments, the i‐motif enabled linking a pair of aptamer fragment‐bearing TDNs into a dimeric split‐aptamer‐based ATP aptasensor,^[^ ^7^ ^]^ or deforming a DTP structure to subsequently dissociate a G‐quadruplex‐based ATP aptasensor.^[^ ^8^ ^]^ Despite their precise operation, however, DNA nanoassemblies generally require extensive computational modeling of the secondary structures and the tertiary folding of multiple DNA strands, which essentially limits design generalizability across different aptamers.^[^ ^9^ ^]^ Moreover, the duplexed DNA structures often impose a design restriction for locating additional functional DNA strands such as cell‐targeting aptamers. Practical challenges arise in their fabrication that usually requires a large quantity of multiple DNA strands through a series of annealing steps with precise stoichiometric control, as well as the stability issues of the probes during an elongated incubation under physiological conditions. In this regard, there are still unmet needs for a high‐throughput strategy to create a ratiometric intracellular DNA aptasensor that is biologically deliverable, robust, and able to be fabricated in a facile manner.

An emerging class of nucleic‐acid‐based structures, rolling circle replication (RCR)‐driven RNA/DNA micro‐/nanoparticles,^[^ ^10^, ^11^ ^]^ offers a potential route to address the challenges. The RCR, including rolling circle amplification (RCA) or rolling circle transcription (RCT), is an isothermal enzymatic technique, in which an RNA or DNA polymerase produces polymeric nucleic acids by repeating complementary copies of a customized template.^[^ ^12^ ^]^ During the process, it spontaneously renders densely packed, flower‐shaped DNA/magnesium pyrophosphate (Mg_2_PPi) hybrid composites via nucleic‐acid‐driven crystallization. Exploiting its template programmability, unique hierarchical structure, and stability against serum/nuclease degradation, the composites have shown wide‐ranging potential, especially for bioimaging^[^ ^13^, ^14^, ^15^ ^]^ and delivery of bio/chemical molecules (e.g., bioactive protein,^[^ ^16^, ^17^, ^18^ ^]^ gene,^[^ ^18^, ^19^, ^20^ ^]^ and drug molecules^[^ ^13^, ^14^, ^21^, ^22^ ^]^). Despite their promise for multifunctional platform design, to the best of our knowledge, the programmable RCR‐based structures have not yet been explored for biosensing platforms that are capable of target‐responsive signaling.

In this study, we present a tumor‐targetable, ratiometric intracellular aptasensing platform that employs the systematic design of RCA‐driven DNA–inorganic hybrid nanocomposites and enables quantitative interpretation of changes in cellular analytes. The flower‐shaped, nanosized DNA particles (termed as nDNF) were spontaneously packaged using long polymeric DNA amplicons empowered by enzymatic amplification. Harnessing a rationally designed multielement‐encoded template, a large quantity of functional DNA strands is produced and self‐compacted into DNA particles that present three major sequence‐driven functionalities, such as a cell‐targeting aptamer, a ratiometric aptasensor, and a cholesterol‐decorating element (**Figure** **1**a,b). For ratiometric fluorescence sensing with single excitation and dual emission, we adapted structure‐switching signaling aptamers into a tripartite design that produce FRET‐based signal readouts, mediated by Cy3 (a donor dye) and Cy5 (an acceptor dye) pairs.^[^ ^23^, ^24^ ^]^ In that scenario, the acceptor dye‐labeled signaling strands are readily disassociated in the presence of targets, and the corresponding FRET signal changes can be directly recorded from the nDNF construct. The tumor targetability of the construct arises from the encoded cell‐targeting aptamers that selectively bind to cell membrane receptors and facilitate subsequent internalization into target cells. As nucleic acids present a highly anionic charge, an additional condensation method using synthetic polycations (e.g., polyethylenimine or poly‐l‐lysine) has been often needed to effectively facilitate the cellular delivery of genetic materials.^[^ ^13^, ^16^, ^19^, ^21^ ^]^ Due to possible restrictions of aptamer functionality caused by nonspecific physisorption of such cationic polymers, we investigated an alternative approach of decorating nDNF with cholesterol via in situ hybridization—by which cholesterol can synergistically enhance cellular uptake of nDNF while minimizing any unwanted interference. As a proof‐of‐principle, we illustrated the targeted delivery of our probe to LNCaP human prostate adenocarcinoma cells and demonstrated ratiometric aptasensing triggered by ATP molecules present in the cells (Figure 1c). To the best of our knowledge, this represents the first demonstration of a biomimetic crystallization‐driven DNA nanosensor that enables ratiometric intracellular detection through target‐triggered signaling. In this exemplar system, we explored the feasible interpretation of fold‐changes in the abundance of cellular analytes beyond the one‐to‐zero type of logic operation or qualitative interpretation. This work paves the way for interrogating cellular analytes whose changes in abundance are rather explained as a continuous function. Established on sequence‐encoded functionalities and RCA‐driven self‐assembly, our proposed platform represents a highly versatile and facile strategy to construct an intracellular ratiometric aptasensor, whereby the design can be readily tailored for wide‐ranging target cell and analyte systems.

**Figure 1:**
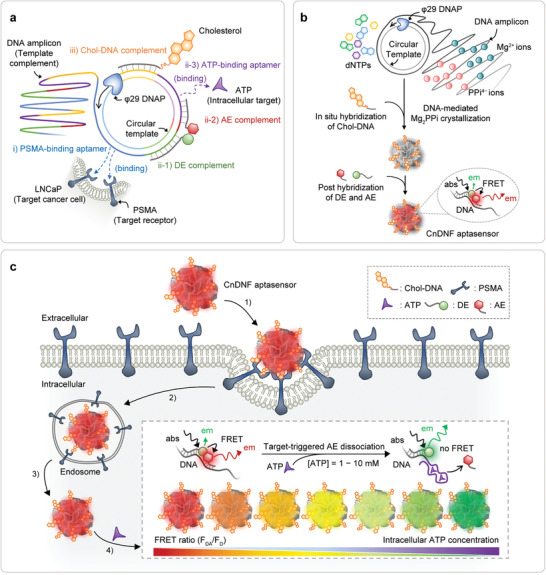
Schematic illustration of an intracellular ratiometric aptasensor. a) Design of multielement‐encoded DNA template used for rolling circle amplification (RCA) reaction. The template DNA complement features three main elements: i) cell‐targeting aptamer, ii) adenosine triphosphate (ATP) FRET‐aptasensor, and iii) cholesterol‐labeled single‐stranded DNA (Chol‐DNA) binding element. φ29 DNAP produces elongated DNA strands with repeating copies of the complementary sequence of the template. b) Fabrication of cholesterol‐decorated DNA nanoflower (CnDNF) aptasensors using a two‐step approach. Chol‐DNA is first incorporated in situ during RCA, producing CnDNF. Donor and acceptor‐labeled DNA elements (DE and AE) are subsequently hybridized into a FRET pair within CnDNF constructs. c) Overview of the working principle of a CnDNF aptasensor. 1) The CnDNF aptasensor binds to the target receptor (prostate‐specific membrane antigen, PSMA) in the cellular membrane and 2) undergoes receptor‐mediated internalization into target cells (LNCaP). 3) Upon entering the cytosol, 4) intracellular target (ATP) recognition events trigger the liberation of AE from the CnDNF aptasensor, enabling FRET‐based ratiometric ATP level monitoring. The illustrations are not to scale.

Since the repeating unit of the RCA products is complementary to the circular template DNA, RCA allows tailor‐designed sequences of the resulting DNA amplicons, including, e.g., DNA aptamers, specific sequences for the incorporation of drug molecules (i.e., GC‐rich for doxorubicin), or DNA probes. We achieved this in our aptasensor platform using designed template DNA to yield elongated DNA strands with multiple units composed of two aptamer sets (which are specific to ATP and prostate‐specific membrane antigen (PSMA), and serve as an aptasensor and a cell‐targeting ligand, respectively) and one binding site for the cholesterol‐labeled DNA strand (Chol‐DNA) (Figure 1a, see Table S1, Supporting Information, for details of DNA sequences). Initially, the ATP aptasensor comprises three domains, including a consensus ATP aptamer sequence^[^ ^23^, ^25^ ^]^ and two binding sites for both FRET‐paired DNA probes. We refer to the donor dye (Cy3)‐coupled DNA strand as the “DE” and the acceptor dye (Cy5)‐labeled DNA strand as the “AE”. Once bound to the amplified DNA strands produced by RCA, the DE and AE are situated in close proximity enabling FRET to occur, where the AE partially occupies a specific portion of the ATP‐binding aptamer promoting its target‐triggered dissociation. Second, the PSMA aptamer—that is selected to bind to a particular transmembrane protein overexpressed in prostate cancer cells^[^ ^26^ ^]^—enables cell targeting and efficient internalization of the cargos through an endocytic pathway.^[^ ^27^, ^28^ ^]^ Finally, there is a growing number of studies showing that cholesterol‐anchored nanostructures can reversibly associate with and laterally diffuse on lipid bilayers of the plasma membrane.^[^ ^29^ ^]^ Given this, we speculated that the decoration of DNF with hydrophobic cholesterol^[^ ^30^ ^]^ would mimic the amphiphilic nature of the cell membrane,^[^ ^29^, ^31^ ^]^ leading to a synergistic effect in cellular uptake together with the receptor‐binding aptamers.^[^ ^32^ ^]^ Importantly, this method offers a route to avoid the condensation of DNA with cationic polymers by electrostatic physisorption as this approach can possibly restrict the sequence availability of target‐/cell‐binding aptamers, interfere with the formation of binding‐competent state of aptamer, and hinder the target molecule's ability to access to the corresponding aptamer.

The CnDNF aptasensor is prepared in a two‐step approach (Figure 1b). First, to synthesize DNA flowers (DNF), a typical RCA was performed, as described previously (Figure S1a, Supporting Information).^[^ ^10^, ^17^ ^]^ As the DNA synthesis proceeds over time, the released pyrophosphate ions (PPi^4−^) are instantly stabilized by counterions, a sufficient amount of magnesium ions (Mg^2+^) supplied by the reaction buffer, rather than being further hydrolyzed to inorganic phosphate (Pi). This means that the concentration of magnesium pyrophosphate (Mg_2_PPi) crystals governing the size and morphology of the DNF will gradually increase over time until all the free Mg^2+^ ions are consumed. The simultaneous precipitation of Mg_2_PPi inorganic crystal phases serves as an essential self‐condensing mechanism, in which the resulting DNA amplicons can be packaged into densely packed particulates of defined size and morphology, rather than remaining as long polymeric strands in solution. To investigate the morphology and size of the DNF at different time points, we conducted scanning electron microscopy (SEM) imaging and dynamic light scattering (DLS) measurements (Figure S1b–d, Supporting Information). Note that the obtained RCA products are purified using centrifugal separation and washed with nuclease‐free water at least three times to remove residual traces such as salts, nucleotides, enzymes, etc. Discrete particles with nano‐rough surface of 200–300 nm in size (termed “nDNF”) appeared after 6 h of reaction and grew into relatively monodisperse, 1–2 µm sized flower‐like spherical microparticles (termed “μDNF”) after 8 h up to 20 h of incubation, which is consistent with previous reports.^[^ ^17^ ^]^ To aim for cellular internalization, we chose the nDNF prepared with 6 h of incubation time and used in subsequent studies. In order to decorate the nDNF with cholesterol molecules, single‐stranded DNA with cholesterol modification at the 3'‐end (Chol‐DNA) was added to the RCA mixture in various concentrations (1 × 10^−6^, 2 × 10^−6^, 5 × 10^−6^ m, termed CnDNF‐1, CnDNF‐2, and CnDNF‐5, respectively) so that Chol‐DNA can rapidly hybridize to the growing DNA strands in situ during RCA. Next, the resulting CnDNF were annealed with the DE and AE in a hybridization buffer to obtain CnDNF aptasensors. By performing the two‐step approach consisting of spontaneous incorporation of Chol‐DNA during RCA and post‐annealing of DE and AE, we sought to reduce the complexity of the hybridization system involving multiple different strands and maximize the overall hybridization efficiency in a desired sequence‐specific manner. It is important to note that the annealing step is required to ensure efficient hybridization of the dye‐labeled DNA strands, and more importantly, to aid the folding of both PSMA and ATP aptamers into their appropriate conformations.

In order to closely investigate the effect of Chol‐DNA on the morphology and structure, we analyzed the CnDNF series synthesized with the addition of various concentrations of Chol‐DNA (CnDNF‐1, CnDNF‐2, and CnDNF‐5) and compared to nDNF using high‐resolution transmission electron microscopy (HR‐TEM), SEM, and DLS (**Figure** **2**a and Figure S2, Supporting Information). Regardless of the amount of Chol‐DNA added, the SEM and HR‐TEM images showed no appreciable difference in size nor morphology of the particles within the 6 h of the reaction time. The relatively narrow DLS intensity distribution (PDI ≈ 0.14) confirmed that fairly monodisperse and spherical particles were obtained (Figure S3a, Supporting Information). The Z‐average hydrodynamic diameters of nDNF and CnDNF were measured to be 350–450 nm, which are consistent with the sizes observed by SEM and TEM considering the swelling of polymeric DNA amplicons in an aqueous environment (Figure 2b). While very marginal differences were detected by SEM and TEM, we observed a small, but evident decrease in the hydrodynamic diameter when Chol‐DNA was added above 2 × 10^−6^ m, showing size reduction by 17.0% for CnDNF‐5. We noted that this size reducing effect by Chol‐DNA appeared more significant after an extended RCA reaction time, as shown in CμDNF sets (20 h RCA) (Figure S4, Supporting Information). We also observed that UV absorbance spectra of nDNF and CnDNF showed marginal differences, whereas DNA content was considerably decreased in the presence of Chol‐DNA, up to 0.76‐fold reduction for CnDNF‐5 with respect to nDNF (Figure S3b,c, Supporting Information). We hypothesize that these differences could be attributed to: 1) Chol‐DNA spontaneously bound to the newly synthesized DNA strands and drove aggregation via hydrophobic interaction, leading to more densely packed, smaller DNA constructs, and 2) hydrophobic interaction with a hydrophobic domain of ϕ29 DNA polymerase (ϕ29 DNAP) during RCA might have slowed down the kinetics of the enzymatic reaction. To quantify the cholesterol incorporation, we used Cy3‐labeled Chol‐DNA at the 5'‐end (termed Cy3‐Chol‐DNA) instead of Chol‐DNA and followed the same CnDNF fabrication procedures (Figure 2c). The amount of cholesterol within the constructs was indirectly measured from the fluorescence signals based on the calibration curve using known concentrations of Cy3‐Chol‐DNA (Figure S3d,e, Supporting Information). While no detectable amount was observed from nDNF, the calculated concentration of Cy3‐Chol‐DNA (per 200 ng µL^−1^ of DNA of CnDNF) corresponds to a molar fraction of 5.31%, 11.2%, and 18.5% for CnDNF‐1, CnDNF‐2, and CnDNF‐5, respectively, out of the total DNA contents.

**Figure 2:**
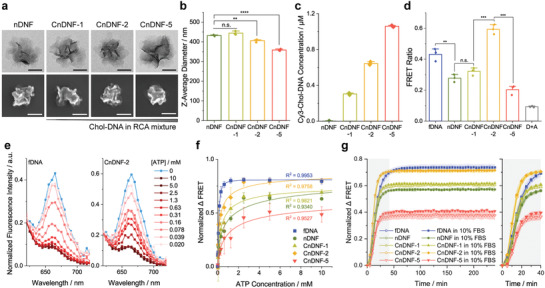
Characterization of CnDNF aptasensor. a) Representative TEM (top) and SEM (bottom) images of nDNF and CnDNF fabricated with varying Chol‐DNA concentrations (1 × 10^−6^, 2 × 10^−6^, and 5 × 10^−6^ m): nDNF, CnDNF‐1, CnDNF‐2, and CnDNF‐5. Scale bar, 100 nm (top) and 200 nm (bottom). b) Z‐average diameter measured by DLS. c) Calculation of concentration of Cy3‐Chol‐DNA in nDNF and CnDNF (per 200 ng µL^−1^ of DNA). d) FRET ratios of nDNF, CnDNF, and fDNA aptasensors (FRET ratio = FI_DA_/FI_D,_ where FI_DA_ and FI_D_ refer to acceptor emission maximum and donor emission maximum, respectively). e) Emission fluorescence spectra (excitation at 520 nm) of fDNA and CnDNF‐2 normalized with respect to FI_DA_ with varying concentrations of ATP. f) Dose‐responsive ratiometric signal changes of nDNF, CnDNF, and fDNA with varying concentrations of ATP. The signal changes are defined as FRET ratio change (ΔFRET = FRET_0_ − FRET_ATP_, where FRET_ATP_ and FRET_0_ are FRET ratio in the presence and absence of a target, respectively), normalized with respect to FRET_0_. Each profile was fitted with a sigmoidal curve (Hill fit) with the *R*‐squared (*R* ^2^) indicated. g) Ratiometric signal changes from the aptasensors as a function of time in the presence and absence of FBS (10% v/v) at 37 °C after the addition of 2.5 × 10^−3^ m of ATP. Prior to the measurement, the aptasensors were incubated at 37 °C for 4 h in each solution. The signal changes are defined as ΔFRET normalized to FRET ratio at 0 min‐timepoint (FRET_0min_) immediately after the addition of ATP. d–f) Fluorescence measurements were carried out after 1 h of incubation at 37 °C and recorded at 37 °C. Data represent mean ± s.d. or mean for three repeated measurements in (b) or three independent experiments (c–g). n.s. (not significant, *p* > 0.05), ****p < 0.0001, ***p < 0.0001, **p < 0.01, and *p < 0.05 based on one‐way analysis of variance (ANOVA) and Tukey's honest significance test.

Our proposed ratiometric nanosensor operation is based on the changes from a high‐FRET to low‐FRET configuration, triggered by the target binding and the subsequent liberation of the AE. To study the feasibility of the design, solution‐based free DNA aptasensor (fDNA) using a capture probe (CP) was assessed as a simple, 1D “free” analog (Figure S5, Supporting Information). It has been commonly understood that aptamers present unique secondary structures that maintain an intrinsic affinity to a specific ligand (target), referred to as a binding competent conformation.^[^ ^24^ ^]^ We first screened buffer conditions capable of promoting high folding stability of the aptamer (in a binding‐competent state), retaining the AE hybridization affinity, and supporting the target‐triggered AE dissociation (Figure S5a,b, Supporting Information). It has been understood that both monovalent sodium ions (Na^+^) and divalent magnesium ions (Mg^2+^) are essential to facilitate the correct folding and retention of a binding‐competent state of the aptamers,^[^ ^33^, ^34^ ^]^ which was demonstrated by the loss of target‐responsiveness in the absence of metal cations. We observed that Mg^2+^‐containing buffers produced a relatively higher signaling magnitude compared to Na^+^‐containing, single composition buffer. This is in agreement with previous studies that Mg^2+^ plays a key role in the formation of the two‐stacked G‐quartet structure and the associated ligand‐binding pockets of the ATP‐binding aptamer,^[^ ^25^, ^34^, ^35^ ^]^ which is crucial for both the binding affinity and the structural integrity of the ligand‐bound states.^[^ ^33^ ^]^ Therefore, we selected the buffer containing both Na^+^ and Mg^2+^ ions (300 × 10^−3^ m NaCl, 5 × 10^−3^ m MgCl_2_, 20 × 10^−3^ m Tris–HCl, pH 7.5) for further assays, where the co‐abundance of both monovalent and divalent metal cations closely resembles that in physiological environments.^[^ ^36^ ^]^ The target selectivity of the designed aptasensor was confirmed by comparing other nucleoside triphosphates, such as uridine triphosphate (UTP), cytidine triphosphate (CTP), and guanosine triphosphate (GTP) (Figure S5c, Supporting Information).

After validating the ratiometric signaling in solution, we investigated the aptasensor operation when composed of the densely packed DNA amplicon within a hierarchical, 3D DNF construct. We first determined the optimal relative ratio of the amounts of AE, DE, and CnDNF (or nDNF) applied to ensure a maximal FRET ratio of the formed aptasensors when no targets are present (Figure S6, Supporting Information). We then evaluated the FRET ratio in the absence of target after 1 h of incubation at 37 °C, which is a critical parameter for our signaling that defines the ground‐level signal and serves as a normalization factor for target‐responsive FRET changes (Figure 2d). A considerable reduction in FRET ratio by 35.7% was observed when the DE and AE were used in nDNF constructs compared to fDNA, indicating fewer FRET pairs were formed within the construct (“immobilized” within the DNF construct) compared to CP (a “free” analog in solution). Notably, we observed increased FRET ratios in CnDNF‐1 and CnDNF‐2 compared to that of nDNF, where the highest FRET ratio was observed in CnDNF‐2. We hypothesize that this could be attributed to the impact of DNA surface density and steric/electrostatic hinderance influencing the surface‐bound DNA hybridization efficiency.^[^ ^37^, ^38^ ^]^ The hybridization of Chol‐DNA with nDNF would presumably increase the rigidity of the neighboring strand to some extent, changing surface‐bound DNA into a more rigid, readily accessible conformation.^[^ ^38^ ^]^ Additionally, the cholesterol molecules in nDNF may serve as hydrophobic backfillers—molecules spatially filling the residual space between the dense DNA strands—reducing local DNA densities near the surface and re‐orienting DNA into a more upright position.^[^ ^39^ ^]^ On the other hand, the significantly decreased FRET ratio of CnDNF‐5 implies that a large amount of Chol‐DNA present in the system likely sterically hindered the hybridization of DE and AE, leading to fewer FRET pairs forming as compared to CnDNF‐2.

In the presence of ATP molecules, the fluorescence spectra showed a significant increase in donor emission while acceptor emission decreased, confirming the successful target‐triggered dissociation of AE from the DNF constructs (Figure 2e and Figure S7a–e, Supporting Information). The FRET ratio changes in the presence of different concentrations of ATP were recorded in a dose–response curve (Figure 2f and Figure S7f–k, Supporting Information), in which reliable ratiometric signaling was achieved regardless of the aptasensor configuration. We noted that, however, the signal‐saturating target concentration and linear detection range appeared to be varied. In particular, the fDNA aptasensor exhibited the narrowest linear detection range in the low concentration regime and the lowest saturating target concentration. On the other hand, nDNF‐based constructs showed substantially wider dynamic ranges of linear detection with signal saturation at a far higher target concentration. This result implies that the target accessibility and binding events were differing significantly between the bound and free cases. Finally, we further validated the successful target‐triggered dissociation of AE from DNF aptasensors with fluorescence correlation spectroscopy (FCS) analysis, where we confirmed over 99% liberation of AE from DNF aptasensors in the presence of ATP (Figure S8, Supporting Information).

To ensure the feasibility of detection in intracellular environments, we initially incubated the aptasensors in 10% (v/v) fetal bovine serum (FBS)‐containing buffer for 4 h at 37 °C, followed by the addition of ATP (2.5 × 10^−3^ m) (Figure 2g). The introduction of ATP caused rapid changes in the FRET ratio, approaching saturation within 40–60 min. During incubation, we observed no appreciable reduction in FRET response regardless of the presence of serum, and the relative differences in FRET signals were in good agreement with the dose–response curves in Figure 2f. We used *t*_1/2_ (defined as the time required to reach the half‐maximal signal)^[^ ^23^ ^]^ to monitor the kinetics of signal generation of each aptasensor. We observed that the fDNA aptasensor exhibited a marginally longer *t*_1/2_ (14.4 min) compared to nDNF‐based aptasensors, and the fastest signal switching was found in the CnDNF‐2 aptasensor (11.0 min). The increase in FRET ratios from nDNF and CnDNFs suggests that although signaling magnitude does depend on the amount of cholesterol, the AE can be readily dissociated from the densely packed aptasensor under physiological conditions in a comparable regime (even slightly faster) compared to the liberation from the CP. Taking such enhanced sensing performance into consideration, including low ground‐level signal, large signaling magnitude, and fast kinetics, we selected CnDNF‐2 (termed CnDNF, hereafter) for further in vitro studies.

Using the ratiometric sensing strategy, we explored the targeted delivery of the CnDNF via receptor‐mediated internalization. As indicated earlier (Figure 1a), our sensing platform provides intrinsic targetability via PSMA‐binding aptamers (Apt), thereby requiring no further post‐functionalization. For a systematic study, we chose a PSMA‐positive, androgen‐dependent prostate cancer cell line (LNCaP) and a PSMA‐null, androgen‐independent cell line (PC3).^[^ ^28^ ^]^ The cells were cultured under the same conditions (i.e., culture media supplemented with 10%, v/v FBS and 1%, v/v penicillin‐streptomycin).

In a typical procedure to evaluate selective uptake and association of the probes, LNCaP or PC3 cells were incubated with DE‐hybridized (Cy3‐labeled) CnDNF (or nDNF) at 37 °C for 2 and 24 h, respectively. The Cy3‐positive population was then determined based on the single cell population using flow cytometry (**Figure** **3**a–d and Figure S9, Supporting Information). To ensure that sufficient fluorescent signals are readable using flow cytometry, 60 × 10^−9^ to 240 × 10^−9^ m of nDNF and CnDNF were used for the incubation. Overall, a significantly higher Cy3‐positive population was observed for both nDNF and CnDNF in LNCaP compared to PC3 (Figure 3b), which demonstrates the selective binding and association with PSMA‐expressing cells. To be specific, we observed 46.3% and 85.2% of Cy3‐positive LNCaP cells when exposed to CnDNF for 2 and 24 h, respectively. This corresponds to an 3.8‐fold higher uptake efficiency at 2 h compared to that of PC3 cells. A higher population was observed for CnDNF compared to nDNF in both cells, which indicates cholesterol‐decoration‐enhanced cellular uptake. To further examine the effect of cholesterol, we designed a scrambled template with the same length as the Apt‐presenting template but of randomly ordered sequences (Scram) to be irrelevant to the targeting‐binding aptamers. When exposed to the Scram‐bearing CnDNF, a substantially decreased Cy3‐positive LNCaP population (88.8% of reduction after 2 h with respect to Apt‐bearing CnDNF) was measured, suggesting that the LNCaP's uptake efficiency of CnDNF is predominantly mediated by Apt binding (Figure 3c). These findings are likely a synergistic function of passive attraction to the cellular membrane by cholesterol and active targeting using the receptor‐specific binding of the aptamer. We observed a concentration‐dependent increase of Cy3‐positive LNCaP cells reaching 81.4% for 240 × 10^−9^ m of DNA after 2 h of treatment, which is in good agreement with previous reports on the concentration‐dependence of active targeting (Figure 3d).^[^ ^32^ ^]^ We also observed no apparent cytotoxic effects when a series of concentrations of nDNF and CnDNF were incubated with LNCaP cells at 37 °C for 24 h (Figure 3e).

**Figure 3:**
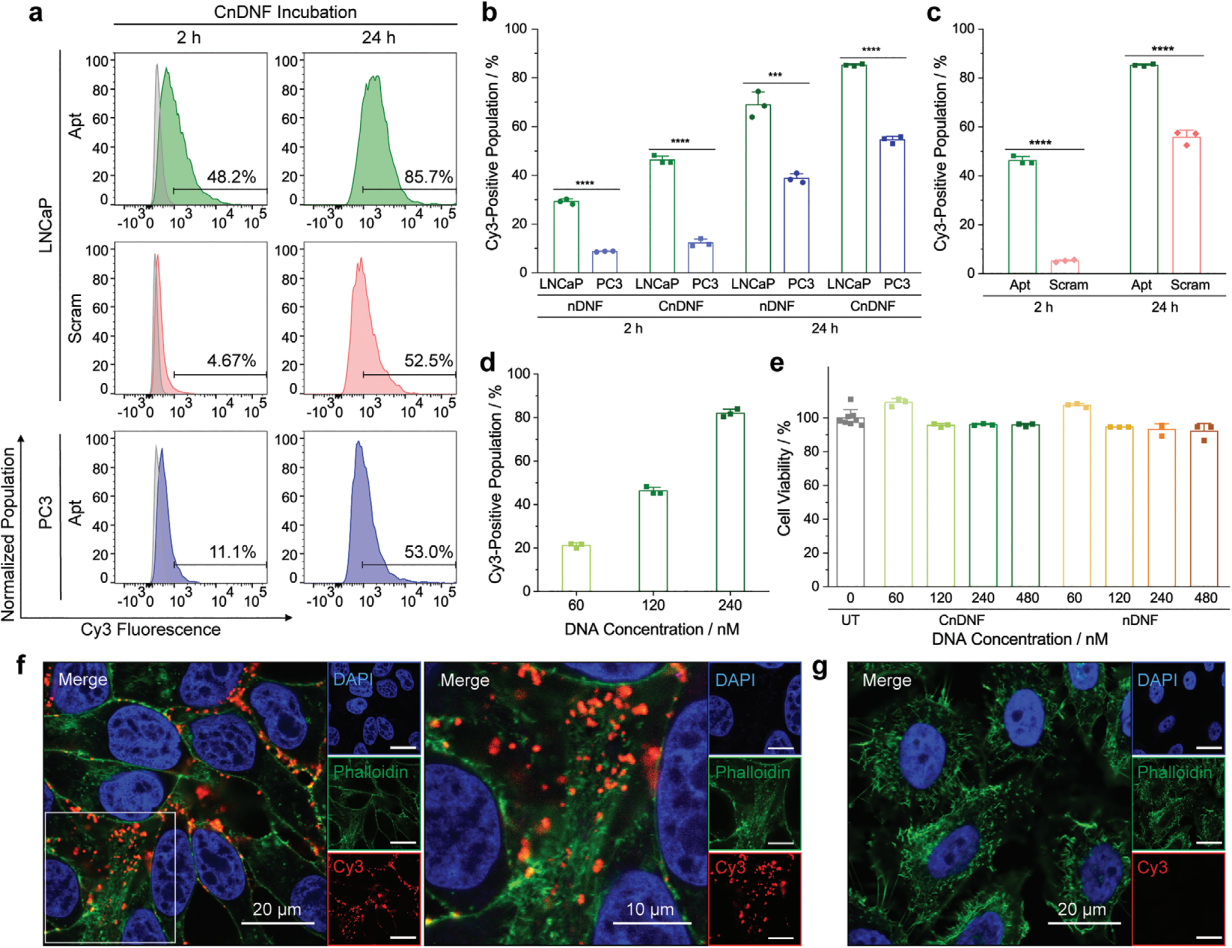
Cellular uptake of CnDNF. a) Representative histograms of cell population versus Cy3 fluorescence intensity measured by flow cytometry analysis. The cells were treated with Cy3‐labeled CnDNF (120 × 10^−9^ m) at 37 °C for 2 and 24 h. PSMA‐binding aptamer (Apt) and scrambled aptamer (Scram)‐producing templates were used to validate the selective internalization of the CnDNF. Percentage values represent the Cy3‐positive population of particle‐treated cells. The gray profiles show cell populations without particle treatment. b) Comparison of the Cy3‐positive population of LNCaP and PC3 cells incubated with nDNF and CnDNF (120 × 10^−9^ m) at 37 °C for 2 and 24 h, respectively. c) Cy3‐positive population of LNCaP cells exposed to CnDNF presenting Apt and Scram at 37 °C for 2 h. d) Concentration dependency of the Cy3‐positive population of LNCaP cells treated with CnDNF of varied concentrations of DNA (60 × 10^−9^, 120 × 10^−9^, and 240 × 10^−9^ m) at 37 °C for 2 h. e) Viability of LNCaP cells after treatment with varying concentrations of nDNF and CnDNF for 24 h, normalized with respect to untreated cells (UT). The bars represent mean ± s.d. for untreated cells (*n* = 8) and particle‐treated cells (*n* = 3). f,g) Representative confocal microscopy images of cellular internalization of Cy3‐labeled CnDNF (red): f) LNCaP and g) PC3 cells. The cells were incubated with CnDNF (120 × 10^−9^ m) at 37 °C for 2 h, and then counterstained with DAPI (4′,6‐diamidino‐2‐((phenyl‐indole)), blue, nucleus) and Alexa Fluor 488‐labeled phalloidin (green, F‐actin). Apt‐bearing nDNF and CnDNF were used unless otherwise stated. Data in (b–d) represent mean ± s.d. for three independent experiments. ****p < 0.0001, ***p < 0.001 based on one‐way ANOVA and Tukey's honest significance test.

We further employed confocal microscopy to corroborate the selective internalization and to visualize intracellular distribution of CnDNF (Figure 3f,g). After 2 h of incubation, we observed the appearance of red fluorescence corresponding to Cy3‐labeled CnDNF in LNCaP cells, while no appreciable cellular uptakes were observed in PC3 cells. In particular, the defined particulate fluorescence suggests that the fluorescence was not likely originated from dissociated fluorophores but from the probes that remained relatively topologically intact.^[^ ^17^ ^]^ To verify whether they were confined in intracellular organelles, a co‐localization study was performed by sequential incubation of CnDNF for 2 h followed by additional 2 h of staining with LysoTracker, a low‐pH indicator that accumulates within late endosomes and lysosomes^[^ ^40^ ^]^ (Figure S10, Supporting Information). The images showed that most of the CnDNF were found not to be co‐localized with acidic compartments, while some of them ended up showing merged fluorescence signals—meaning some of the internalized particles underwent the endocytic pathway and were entrapped in lysosomes. It is also noteworthy that the marked increase in fluorescence intensity measured by flow cytometry after 24 h of the incubation (Figure 3b and Figure S9g, Supporting Information), by implication, supports the explanation that the majority of probes were not digested nor secreted into extracellular environments during endocytosis, but rather gradually accumulated in cytosolic regions. Nonetheless, considering the co‐existence of the cholesterol and receptor‐binding aptamer, along with unique physiochemical properties of CnDNF (e.g., nonsmooth surface topology and the presence of inorganic elements), its endocytic pathway may well be complicated, where additional in‐depth studies will need to follow.

Having successfully demonstrated targeted delivery, we sought to explore the feasibility of ratiometric sensing in intracellular environments using CnDNF aptasensors. We employed confocal microscopy with a specific laser set‐up to excite Cy3, followed by a sequential recording of the emission profiles through two independent channels for the donor (Cy3) and acceptor (Cy5). To test their capability of monitoring intracellular ATP level changes, the cells were pre‐treated with drugs that either inhibit or induce intracellular ATP production for 4 h, followed by incubation with the CnDNF aptasensors for 2 h before imaging. In particular, we selected oligomycin (OM), which is a specific inhibitor of the ATP synthase,^[^ ^41^ ^]^ and etoposide (EP), a chemotherapy drug that induces cytosolic ATP by apoptotic stimulation.^[^ ^42^ ^]^

**Figure** **4**a shows the representative confocal microscopy images of internalized CnDNF aptasensors, in which both the red fluorescence from Cy5 (FRET channel) and green fluorescence from Cy3 (donor channel) were obtained. Taking a ratiometric approach, the average fluorescence intensities of donor and FRET channel pixels (FI_D_ and FI_DA_) were calculated across multiple images. The normalized proportion of intensity from each channel out of the total fluorescence (FI_Total_) showed that the aptasensors internalized in OM‐treated LNCaP cells exhibited increased FI_DA_ and reduced FI_D_ compared to untreated LNCaP, whereas EP‐treatment yielded the opposite results (Figure 4b). In a ratiometric approach, we calculated the FRET ratio of FI_DA_ to FI_D_ for the target‐responsive signal. Normalized with respect to results from the control (untreated), the FRET ratio clearly showed an evident increase (by 17.0%) and a decrease (by 8.00%) of ratiometric signals in OM‐ and EP‐treated LNCaP cells, respectively (Figure 4c). To confirm the relative ATP changes observed from our approach, the cellular ATP levels were quantified from lysed cells using a commercially available luminescence‐based assay (Figure S11, Supporting Information). We confirmed that the ATP concentration in the lysed LNCaP cells decreased after the OM treatment (82.7% compared to control) while a slight increase was observed in EP‐treated cells (105% compared to control), whereby the relative fold‐changes were markedly consistent with our results from the aptasensing. As shown in the intracellular ATP level as an exemplar, changes in concentration of cellular analytes are often of continuous function that cannot be defined as one‐to‐zero type of operation (presence or absence) and are rather explained in a relative fold‐change. In this regard, our approach demonstrates a potential platform that enables ratiometric detection of relative up‐ and down‐regulation of endogenous biomolecules in a quantitative manner.

**Figure 4:**
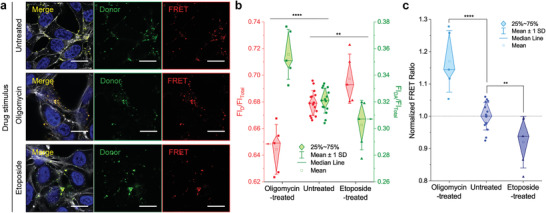
Intracellular ratiometric detection using selectively internalized CnDNF aptasensors. a) Representative confocal microscopy images of CnDNF aptasensors internalized to LNCaP cells after 2 h of incubation at 37 °C. Fluorescence of CnDNF was recorded through two independent emission channels: donor (green, Cy3) and FRET (red, Cy5). To alter the intracellular ATP level, LNCaP cells were pretreated with oligomycin (200 µg mL^−1^) or etoposide (200 × 10^−6^ m) for 4 h at 37 °C, followed by CnDNF incubation. The cells were counterstained with DAPI (blue, nucleus) and Alexa Fluor 488‐labeled phalloidin (white, actin). Scale bar: 20 µm. b) Relative changes of CnDNF fluorescence intensity from each channel in drug‐treated LNCaP cells. The average fluorescence intensities of donor and FRET channel pixels (FI_D_ and FI_DA_) were normalized to total fluorescence intensity (FI_Total_ = FI_DA_ + FI_D_). c) Quantitative analysis of ATP level changes by calculating the FRET ratio from the obtained images (FRET ratio = FI_DA_/FI_D_), normalized with respect to results from untreated cells. Box plots represent results from untreated (*n* = 15 images) and drug‐treated (*n* = 5 images) LNCaP cells. ****p < 0.0001, **p < 0.01 based on one‐way ANOVA and Tukey's honest significance test.

In this work, we developed cholesterol‐anchored DNA nanoflower probes that enable FRET‐based ratiometric intracellular aptasensing upon selective internalization into target cells. Empowered by RCA‐driven self‐assembly of DNA nanoflowers, our strategy provides a simple and efficient method to construct a multifunctional intracellular aptasensor nanoprobe. The probes present inherent biocompatibility, structural integrity, efficient cellular uptake, and sequence‐driven multifunctionalities, without the need for additional nanoparticle carriers nor extensive computational modeling of DNA structures for operation. As a proof‐of‐principle, we carefully designed a template DNA that consists of three distinctive sets of DNA sequences, including a PSMA‐binding aptamer, an ATP‐binding aptamer, and a cholesterol‐binding element. We demonstrated that the particle size and FRET signals of the aptasensor can be controllably regulated by introducing Chol‐DNA strands. The enhanced internalization efficiency of the probe was achieved in PSMA‐rich LNCaP cells, which can be attributed to cholesterol decoration as a potential route that worked in a synergistic way without restricting DNA sequence availability. Finally, we achieved quantitative FRET‐based ratiometric aptasensing from the selectively delivered probes, demonstrated by the responsive FRET signals when changing intracellular ATP levels with two drugs. Exploiting the intrinsic programmability of template DNA by encoding multifunctional elements, this designer probe highlights a versatile strategy by simply modifying the template sequences with other aptamers without altering any fabrication step nor sensing operation. Given the fundamental importance of monitoring abundance of endogenous biomolecules during biological processes or drug treatments, our versatile platform will open up myriads of bioanalytical possibilities as a robust biologically deliverable toolkit that enables probing of dynamic changes in biological profiles in a ratiometric and quantitative manner.

Supporting Information

Supporting Information is available from the Wiley Online Library or from the author.

## Conflict of interest

The authors declare no conflict of interest.

## Supporting information

Supporting Information

## Data Availability

The data that support the findings of this study are available from https://doi.org/10.5281/zenodo.4436438.
