# Supplementary material for: Tumor‐Targeting Cholesterol‐Decorated DNA Nanoflowers for Intracellular Ratiometric Aptasensing
Source: Adv Mater. 2021 Feb 8;33(11):2007738. doi: 10.1002/adma.202007738 (PMC7610848; doi:10.1002/adma.202007738)
Supplement: Supplementary file 1 — Supporting Information [file ADMA-33-2007738-s001.pdf]

© 2021 Wiley-VCH GmbH

ADVANCED MATERIALS

Supporting Information

for *Adv. Mater.*, DOI: 10.1002/adma.202007738

Tumor-Targeting Cholesterol-Decorated DNA Nanoflowers for Intracellular Ratiometric Aptasensing

*Nayoung Kim, Eunjung Kim, Hyemin Kim, Michael R. Thomas, Adrian Najer, and Molly M. Stevens**

Supporting Information

For *Adv. Mater.*, DOI: 10.1002/adma.202007738

Tumor-Targeting Cholesterol-Decorated DNA Nanoflowers for Intracellular Ratiometric Aptasensing

*Nayoung Kim, Eunjung Kim, Hyemin Kim, Michael R. Thomas, Adrian Najer, and Molly M. Stevens**

Department of Materials, Department of Bioengineering and Institute of Biomedical Engineering, Imperial College London, SW7 2AZ London, UK

E-mail: m.stevens@imperial.ac.uk

Keywords: ratiometric, DNA flowers, aptasensors, rolling circle amplification, FRET

Experimental Section

Materials: High-performance liquid chromatography purified oligonucleotides were purchased from Integrated DNA Technologies (IA, US) and used without additional purification (see Supplementary Table S1 for details of DNA sequences used). T4 DNA ligase, exonuclease I, phi29 DNA polymerase (φ29 DNAP), and deoxynucleotides (dNTPs) solution mix were obtained from New England Biolabs (UK). Oligomycin from *Streptomyces diastatochromogenes* was purchased from Merck KGaA (Germany). Chill-out™ Liquid Wax (liquid wax) was obtained from Bio-Rad Laboratories (US). Etoposide was purchased from VWR International (PA, US). Adenosine 5'-triphosphate (ATP), cytidine 5'-triphosphate (CTP), guanosine 5'-triphosphate (GTP), uridine 5'-triphosphate (UTP), 4,6-diamidino-2-phenylindole (DAPI), Alexa Fluor™ 488 Phalloidin, Pierce™ methanol-free 16% formaldehyde (w/v) (PFA), Tris-HCl buffer (pH 7.5 and pH 8.0), EDTA solution (pH 8.0), Dulbecco's PBS (DPBS), heat-inactivated fetal bovine serum (FBS, Brazil), RPMI 1640 medium, LysoTracker™ Green DNA-26, Quant-iT™ PicoGreen® dsDNA assay, PrestoBlue™ Cell Viability Reagent were obtained from Thermo Fisher Scientific (MA, US). Luminescent ATP Detection Assay Kit was purchased from Abcam (UK). RNase/DNase-free distilled water (nuclease-free water, Thermo Fisher Scientific, MA, US) was used for the preparation of all aqueous solution, and the Eppendorf RNA/DNA LoBind microcentrifuge tubes were used to maximize the sample recovery.

Preparation of circular template DNA: Circular template DNA was prepared by an enzymatic ligation method reported by Kim *et al*.[1] First, a 5'-phosphorylated linear template DNA (5 μM) was hybridized with a primer (10 μM) in ligase reaction buffer (50 mM Tris-HCl, 10 mM $MgCl_2$, 1 mM ATP, 10 mM DTT, pH 7.5) by heating at 95 °C

for 5 min and slowly cooling down to room temperature over 3 h (approximately at a cooling rate of 0.5 °C min^{-1}). The nick of hybridized DNA was jointly sealed by T4 DNA ligase (20 U μL^{-1}) by incubating at 16 °C overnight, followed by heat inactivation of the enzyme at 65 °C for 10 min (final volume of 100 μL). The ligated template DNA was further treated with exonuclease I (480 mU μL^{-1}) in reaction buffer (67 mM glycine–KOH, 6.7 mM $MgCl_2$, 10 mM β-mercaptoethanol, pH 9.5) at 37 °C for 1.5 h to remove residual primers, followed by heat inactivation of the enzyme at 80 °C for 15 min (final volume of 250 μL). The obtained template DNA (2 μM) was used for rolling circle amplification (RCA) reaction without further purification.

Preparation of cholesterol-decorated DNA nanoflowers (CnDNF): We refer to the DNA flowers as the 'DNF', nano-sized DNF as the 'nDNF', micron-sized DNF as the 'μDNF', and Chol-DNA-decorated nDNF and μDNF as 'CnDNF' and 'CμDNF' in this study. In a typical RCA procedure to synthesize DNF (both nDNF and μDNF), RCA mixture consisted of circular template DNA (0.6 μM), dNTPs (1 mM), and phi29 DNA polymerase (φ29 DNAP, 1 U μL^{-1}) in reaction buffer (50 mM Tris-HCl, 10 mM $MgCl_2$, 10 mM $(NH_4)_2SO_4$, 4 mM DTT, pH 7.5) (total volume of 50 μL). For cholesterol decoration, cholesterol-labeled single-stranded DNA (Chol-DNA) was additionally added to the reaction mixture in different concentrations (1, 2, and 5 μM). The RCA mixture was incubated at 30 °C for 6 h to fabricate nDNF, whereas incubated at 30 °C for 20 h to fabricate μDNF. The RCA reaction was terminated by heat inactivation of φ29 DNAP at 65 °C for 10 min. The RCA products were thoroughly washed with nuclease-free water three times by centrifugation at 5,000 g for 10 min to remove any free proteins and DNA strands. All particle samples were dispersed in nuclease-free water and kept at 4 °C until use.

The amount of DNA amplified during RCA was quantified by PicoGreen dsDNA reagent. Briefly, nDNF or CnDNF were incubated with the PicoGreen reagent in a 96-well plate for 5 min at room temperature, and the fluorescence intensity (excitation 480 nm, emission 520 nm) was measured using an EnVision multilabel plate reader (Perkin Elmer, USA). The DNA concentration was determined from the standard calibration curve of serial dilutions of lambda DNA (λ-DNA) provided by the manufacturer.

In order to hybridize the Cy3-labeled DNA element (DE) and Cy5-labeled DNA element (AE), the purified and quantified CnDNF was added in the hybridization buffer (20 mM Tris-HCl, 300 mM NaCl, 5 mM $MgCl_2$, pH 7.5) with DE and AE. The relative molar ratio (DE:DNF:AE) was 1:1:4, unless otherwise stated. CnDNF molar concentration was determined with respect to the molecular weight of the cholesterol-hybridized complement template, whereas the molecular weight of template complement was used for bare nDNF (and μDNF, where applicable). To form the free DNA aptasensor (fDNA), CnDNF was replaced with a capture probe (CP). The relative molar ratio (DE:CP:AE) was 1:1:4, unless otherwise stated. In order to hybridize DE and AE to DNF (or CP) and anneal the aptamers, the solution was heated at 95 °C for 3 min, followed by slowly cooling down to room temperature over 3 h (approximately at a rate of 0.5 °C min^{-1}). The obtained aptasensor probes were stored at 4 °C and used without further purification.

Characterization of CnDNF: To investigate the morphology and size, the obtained RCA products were imaged by scanning electron microscopy (SEM, Leo Gemini 1525 FEGSEM) in InLens mode at an accelerating voltage of 5 kV. The SEM samples were prepared by drop-casting of each dispersion (2 μL) on a cleaned silicon wafer chip (5

mm × 5 mm) at room temperature, and sputter-coated with 10 nm of chromium using Q150T S sputter coater (Quorum Technologies, UK). High-resolution transmission electron microscopy (HR-TEM, JOEL JEM-2100F) was employed to examine the structure of CnDNF, operating at 200 kV. The TEM samples were prepared by placing 3 μL of the particle dispersion on 200-mesh carbon-coated Cu grids (Electron Microscopy Science, USA). Hydrodynamic diameters of the DNF were analyzed by a Zetasizer Nano ZS (Malvern, UK). UV absorbance spectrum was obtained using a NanoDrop 2000c spectrometer (Thermo Fisher Scientific, UK).

To examine the amount of cholesterol incorporated during RCA, Cy3/cholesterol dual-labeled ssDNA (Cy3-Chol-DNA) was designed. Briefly, Cy3-Chol-DNA was added to the RCA mixture in varying concentrations (1, 2, and 5 μM), and the RCA was performed following the same procedures as described above. The quantity of DNA content in the resulting Cy3-labled CnDNF was determined by using a NanoDrop spectrometer. The fluorescence spectrum (excitation 540 nm) of each Cy3-labeled CnDNF (per 200 μg mL^{-1} DNA) was recorded using an EnVision multilabel plate reader. The DNA concentration was determined using emission maximum at 565 nm and the standard calibration curve of serial dilutions of Cy3-Chol-DNA.

In vitro ATP sensing assay: In a typical procedure, 50 nM of DE and AE hybridized fDNA, nDNF, and CnDNF aptasensors, and varied amounts of concentration of ATP solution (0.02–10 mM) were prepared in assay buffer (20 mM Tris-HCl, 300 mM NaCl, 5 mM $MgCl_2$, pH 7.5) of a final volume 40 μL in half-area 96-well plates. The reaction mixture was sealed and incubated at 37 °C for 1 h. The fluorescence emission was recorded using a multilabel plate reader with excitation at 520 nm (preset at 37 °C, otherwise stated). FRET ratio is defined as the fluorescence intensity ratio of acceptor

(Cy5) emission maximum (FI_{DA}) at 665 nm to donor (Cy3) emission maximum (FI_{D}) at 565 nm (FRET ratio = FI_{DA} / FI_{D}). Target responsive signal is determined by FRET ratio change (ΔFRET = $FRET_0 - FRET_{ATP}$, $FRET_{ATP}$ and $FRET_0$ refer to as FRET ratio in the presence and absence of a target, respectively), normalized with respect to FRET ratio in the absence of a target ($FRET_0$).

Kinetics or target-responsive signal generation were recorded with a SpectraMax M5 microplate reader (Molecular Devices, USA). Briefly, 50 nM of DE and AE hybridized fDNA, nDNF and CnDNF aptasensors, 10% (v/v) FBS (if applicable) were added in assay buffer (20 mM Tris-HCl, 300 mM NaCl, 5 mM $MgCl_2$, pH 7.5) of a final volume 40 μL in half-area 96-well plates. To minimize the evaporation, 100 μL of liquid wax was added above the reaction solution. The plate was incubated at 37 °C for 4 h, followed by the addition of ATP to reach a final concentration of 2.5 mM. Upon the addition, fluorescence emission was measured during the following 4 h with a microplate set at 37 °C, where the FI_{DA} (at 665 nm) and FI_{D} (at 565 nm) was recorded. FRET ratio and ΔFRET are determined in the same manner as described above. To examine the target-responsive signal, ΔFRET normalized with respect to FRET ratio at 0 min-timepoint ($FRET_{0\ min}$) immediately after the addition of ATP.

Fluorescence correlation spectroscopy (FCS): FCS measurements were performed on a commercial LSM 880 (Carl Zeiss, Jena, Germany) equipped with an incubation chamber equilibrated at 37 °C. The following laser was used as excitation source: HeNe-laser for 633 nm (Cy5) using appropriate filter sets to detect the fluorescence signal. Samples were mixed in DNA-low binding tubes and incubated at 37 °C under static conditions. After mixing the sample by pipetting, an aliquot of 5 μL was pipetted onto an ibidi 8-well glass bottom plate. The laser beam was focused

through a 40× C-Apochromat water immersion objective with a numeric aperture of 1.2 and measurements were conducted 200 μm above the glass. The beam waist was calibrated using a 10 nM solution of standard Alexa647 dye (Alexa647: 4.42 × 10^{-6} cm^2 s^{-1}, when corrected for the higher temperature used, 37 °C).[2] Intensity traces of 25 × 5 s (for dyes and free AE) and 25 × 10 s (for DNFs) were recorded, autocorrelated and analyzed. Exported autocorrelation curves from ZEN software (Carl Zeiss, Jena, Germany) were fitted in PyCorrfit program 1.1.6.[3]

Autocorrelation curves presented in the manuscript correspond to the average curves over the whole measurement (125 s and 250 s, respectively), while percentage of free AE and concentration are given for each individual measurement (n = 25). The percentage of free AE was calculated by first fitting the data obtained for a free solution of AE using one component fits ($G_{1comp}(\tau)$) to yield the corresponding diffusion time (τ_1) and brightness for one AE molecule (*CPP1* in kHz). Subsequently, DNF data was fitted with two component fits ($G_{2comp}(\tau)$), while fixing one component to free AE diffusion as obtained above (τ_1). Since bright and slow diffusing DNFs produce a non-proportional contribution to the average autocorrelation curves,[4] overlapping the free AE signal, a correction[5] was incorporated to adjust the fraction of free AE (*F1*) with the accompanying decrease in the number of AE per DNF upon incubation with ATP for all the curves with CPP_{total} > *CPP1* (see % free AE below). A triplet fraction (T) with a triplet time (τ_{trip}) constrained between 1–10 μs was included in all the curves.

$$G_{1comp}(\tau) = \left(1 + \frac{T}{1-T} e^{\frac{-\tau}{\tau_{trip}}}\right) * \frac{1}{N * \left(1 + \frac{\tau}{\tau_D}\right) * \sqrt{1 + \frac{\tau}{SP^2 \tau_D}}}$$

$$G_{2comp}(\tau) = \left(1 + \frac{T}{1-T} e^{\frac{-\tau}{\tau_{trip}}}\right) * \frac{1}{N} * \left[\frac{F_1}{\left(1+\frac{\tau}{\tau_1}\right) * \sqrt{1+\frac{\tau}{SP^2\tau_1}}} + \frac{1-F_1}{\left(1+\frac{\tau}{\tau_2}\right) * \sqrt{1+\frac{\tau}{SP^2\tau_2}}}\right]$$

$$CPP\ total = F1 * CPP1 + (1 - F1) * CPP2$$

$$\#\ DNA - Cy5\ per\ DNF = NR = CPP2/CPP1$$

$$\%\ free\ DNA - Cy5 = \left\{F1 + (1 - F1) * \left(1 - {}^{NR(t)}\!/_{NR(0)}\right)\right\} * 100$$

, where τ_D is the diffusion time (τ_1, τ_2 are diffusion times of each fraction), F_1 is the fraction of component with diffusion time τ_1, N is the effective number of diffusing species in the confocal volume ($N = n1 + n2$), and SP is the structural parameter, which was always fixed to 5. *CPP* corresponds to counts per particle in kHz. *NR(t)* is the number of AE per DNF at timepoint t (with ATP), while *NR(0)* is the number of AE per DNF at the corresponding timepoint in the absence of ATP.

Cell culture: Human prostate adenocarcinoma cell lines (LNCaP and PC3) were provided by the Department of Surgery and Cancer, Faculty of Medicine at Imperial College London, UK. Both cells were cultured in RPMI 1640 medium supplemented with 10% (v/v) FBS and 1% (v/v) penicillin-streptomycin at 37 °C in a humid atmosphere with 5% CO_2. Cell density was determined using a Countess™ II Automated Cell Counter (Thermo Fisher Scientific, UK).

Cellular uptake study: To investigate the cellular uptake and association, DE was hybridized with purified CnDNF or nDNF (µDNF where applicable) following the same procedure as CnDNF aptasensor as described above, resulting in Cy3-labeled DNF. Cells (2×10^5) were seeded in a 12-well tissue culture plate at a seeding density of 2×10^5 cells mL^{-1} and grown into 70–80% confluency. Briefly, 60–240 nM of Cy3-labled CnDNF was incubated with cells in a culture medium supplemented with 1% (v/v) FBS

at 37 °C for varied incubation time (2 h, 4 h, 24 h, where applicable). The cells were then trypsinized, washed with DPBS by centrifugal separation at 1,000 g for 3 min. The pellet was resuspended in 4% (w/v) PFA in DPBS, followed by incubation for 15 min at room temperature. After washing with DPBS, the cells were resuspended in DPBS, filtered through a 40 μm mesh to remove any cell aggregates. All the measurements were performed on a flow cytometer LSRFortessa (BD Bioscience) and data was analyzed using FlowJo™ V10.6.2 software. The obtained histograms of histograms of cell population versus Cy3 fluorescence intensity were normalized to a node.

Cell viability test: LNCaP cells (2×10^4) were seeded in a 96-well tissue culture plate at a density of 2×10^5 cells mL^{-1} and grown into 70–80% confluency. Varied concentrations of nDNF and CnDNF (0–240 nM) were incubated with cells in a culture medium for 24 h at 37 °C. After the removal of free particles, PrestoBlue™ reagent (10×) was 10 times diluted in culture medium and added (100 μL) to each well. After incubation at 37 °C for 24 h, the fluorescence intensity (excitation 560 nm, emission 590 nm) was measured using a SpectraMax M5 microplate reader.

Confocal microscopy: In a typical procedure, cells (5×10^4) were seeded in an ibidi μ-Slid 8-Well Glass Bottom chamber slide (ibidi GmbH, Germany) at a seeding density of 2×10^5 cells mL^{-1}. CnDNF-2 aptasensors prepared with 2 μM Chol-DNA were incubated with the cells in the culture medium with 1% (v/v) FBS at 37 °C for 2 h. The cells were then washed with DPBS and incubated in 4% (w/v) PFA in DPBS for 15 min at room temperature. After washing with DPBS, the cells were permeabilized in 0.1% (v/v) Triton X-100 for 5 min at room temperature, followed by washing again with DPBS. The permeabilized cells then were stained for the actin filaments for 40 min with Alexa

Fluor 488 phalloidin, and subsequently stained for nucleus with DAPI for 5 min and washed with DPBS. For the subcellular localization study, the cells were treated with Cy3-labeled CnDNF (240 nM) for 2 h. Particles were removed, and then the cells were incubated with 100 nM of LysoTracker™ Green DNA-26 dispersed in culture medium for the following 2 h at 37 °C. The cells were washed with DPBS, fixed with 4% (w/v) PFA in DPBS, and stained for nucleus with DAPI. Confocal imaging was performed on a Leica SP5 resonant inverted confocal microscope (Leica Microsystems, Germany).

Intracellular ATP sensing: LNCaP cells (5×10^4) were seeded in an ibidi μ-Slid 8-Well Glass Bottom chamber slide (ibidi GmbH, Germany) at a seeding density of 2×10^5 cells mL^{-1}. To prepare drug-treated LNCaP cells, oligomycin and etoposide solutions were prepared at 200 μg mL^{-1} and 200 μM, respectively, in culture media. The cells were exposed to each drug solution (300 μL) for 4 h at 37 °C. Followed by the removal of free drugs, cells were subsequently treated with CnDNF-2 (120 nM) in the medium containing 1% (v/v) FBS for 2 h at 37 °C. The cells were then washed and prepared for confocal microscopy as described above. Confocal images of CnDNF-2 were obtained using 543 nm laser to excite Cy3, with two emission channels for Cy3 (donor channel) and Cy5 (FRET channel), respectively.

Image analysis was performed using MATLAB (MathWorks, Inc., Natick, MA, USA). To remove cosmic intensity noises, the images were smoothed using a moving-average filter by replacing each pixel value with the average pixel value of neighboring window of 3×3 pixels, including itself. To disregard the effect of varying background intensities, we introduced a threshold model technique where we set a threshold value—which represented the background level for each image—and only took pixel

values above it for further downstream analysis. Taking a quantitative ratiometric approach, the average fluorescence intensities of donor and FRET channel pixels (FI_D and FI_{DA}) were obtained for each image. The total intensity (FI_{Total}) is defined as the sum of FI_D and FI_{DA} ($FI_{Total} = FI_{DA} + FI_D$), and the FRET ratio is determined by the ratio of FI_{DA} to FI_D (FRET ratio = FI_{DA} / FI_D). Image analysis was performed based on batch analysis using raw images without extra modifications using the identical parameters and protocols across frames.

To compare the results based on a commercial kit, the Luminescent ATP Detection Assay Kit was employed, strictly following the manufacturer's protocol. Briefly, LNCaP cells were seeded in a 96-well tissue culture plate at a density of 2×10^5 cells mL^{-1} and grown into confluency. The cells were exposed to each drug solution (100 μL) for 4 h at 37 °C. The provided detergent was then added to lyse the cells and stabilize the ATP, followed by the addition of a substrate containing firefly luciferase and D-luciferin. The generated luminescence was recorded using a multilabel plate reader.

Table S1. Summary of nucleic acid sequences used in this study.

**Name**	**Sequence (5' – 3')**
Linear template DNA[a]	Phosphorylation–ATG AAT ATT ATT ACC TTC CTC CGC AAT ACT CCC CCA GGT AGC GGA GCG TGG CAG G*AG CAT TGC TAT CGT AAG CAG ATG ACC CAA AAC GCA AAA GCG AAA ACG C*TA TTA TGA TA
Scrambled linear template DNA	Phosphorylation–ATG AAT ATT ATT GAG CGT ACT TAG TAG CCT GGA GGA CCT AGC GGA GCG TGG CAG GTT GCG TTG ACT CAA TGT AGC GAT CTA ACT AGT AAC CTA GGC ACT CGC GTA TTA TGA TA
Primer	AAT AAT ATT CAT TAT CAT AAT A
Donor-labeled DNA element (DE)	Cy3–GCG GAG CGT GGC AGG
Acceptor-labeled DNA element (AE)	CTC CCC CAG GT–Cy5
Cholesterol-labeled ssDNA[b] (Chol-DNA)	TGATAATGAATATTA–TEG–Cholesterol
Capture probe (CP)	CCT GCC ACG CTC CGC TAC CTG GGG GAG TAT TGC GGA GGA AGG T
Cy3/cholesterol dual-labeled ssDNA (Cy3-Chol-DNA)	Cy3–TGATAATGAATATTA–TEG–Cholesterol

[a] Note that the underlined sequences in green and italic sequences in blue represent the complementary sequences of adenosine triphosphate (ATP)-binding and prostate-specific antigen (PSMA)-binding aptamers, respectively.
[b] The abbreviation TEG indicates triethylene glycol.

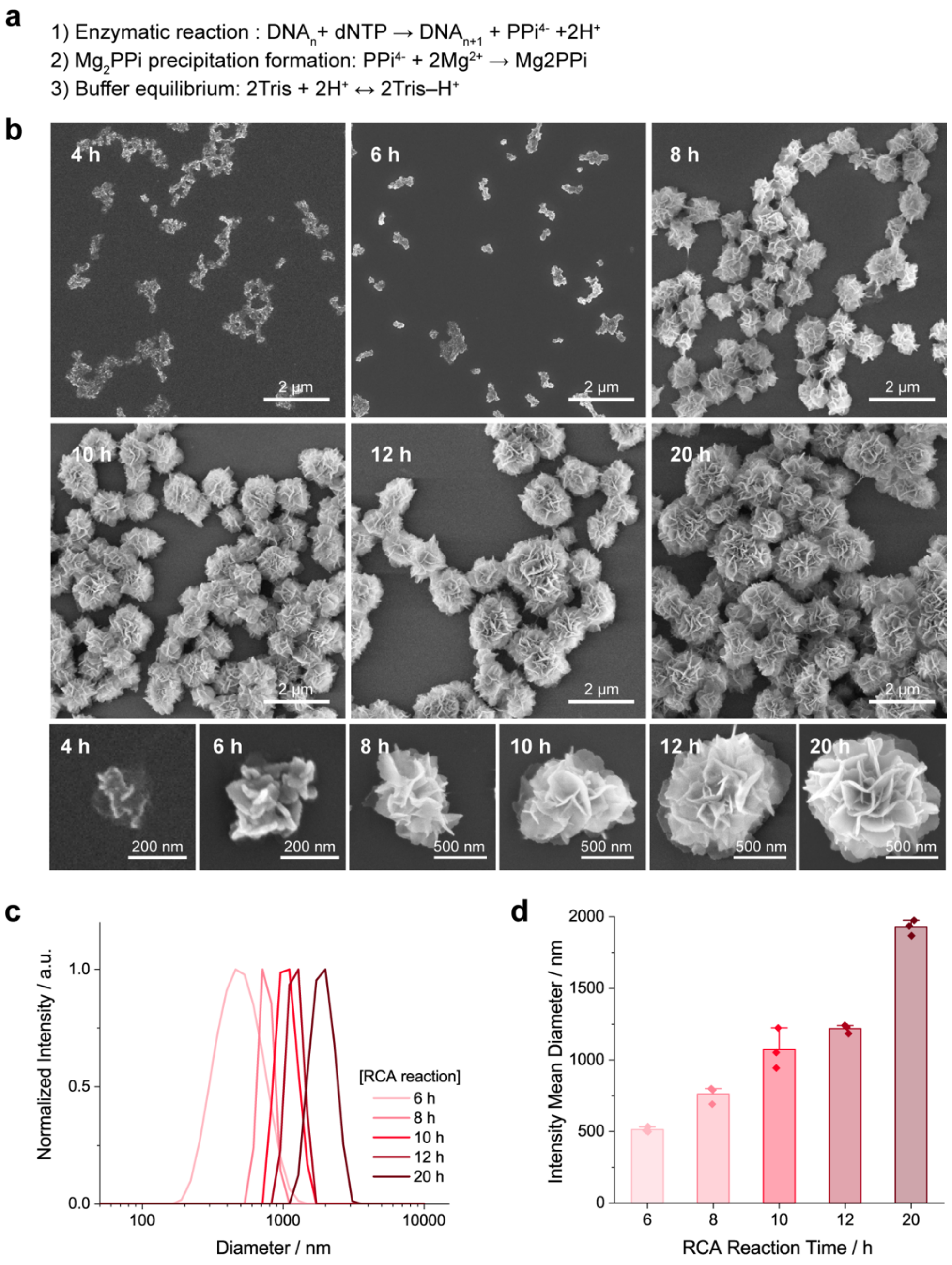

Figure S1. Time-dependent growth of DNF during the RCA process. (a) Three underlying reactions associated in RCA.[1] During the RCA reaction, φ29 DNAP synthesizes new DNA strands by consuming dNTPs, releasing pyrophosphate (PPi^{4-}) ions as one of the by-products from an enzymatic reaction. While the DNA amplicon grows, the enzyme cofactor magnesium (Mg^{2+}) ions that are present in the reaction buffer and the released PPi^{4-} ions precipitate into magnesium pyrophosphate (Mg_2PPi) crystals, which spontaneously self-assembled into DNA–Mg_2PPi hybrid composite particles. The anisotropic liquid-crystallization and the dense packaging of polymeric DNA strands drive noncanonical self-assembly of flower-shaped and sponge-like porous hybrid composites. (b) Representative SEM images of DNF at different RCA reaction time (4–20 h). (c) Intensity particle size distribution and (d) hydrodynamic diameter of DNF at different RCA reaction time measured by DLS. Data represent mean or mean ± s.d. for three repeated measurements.

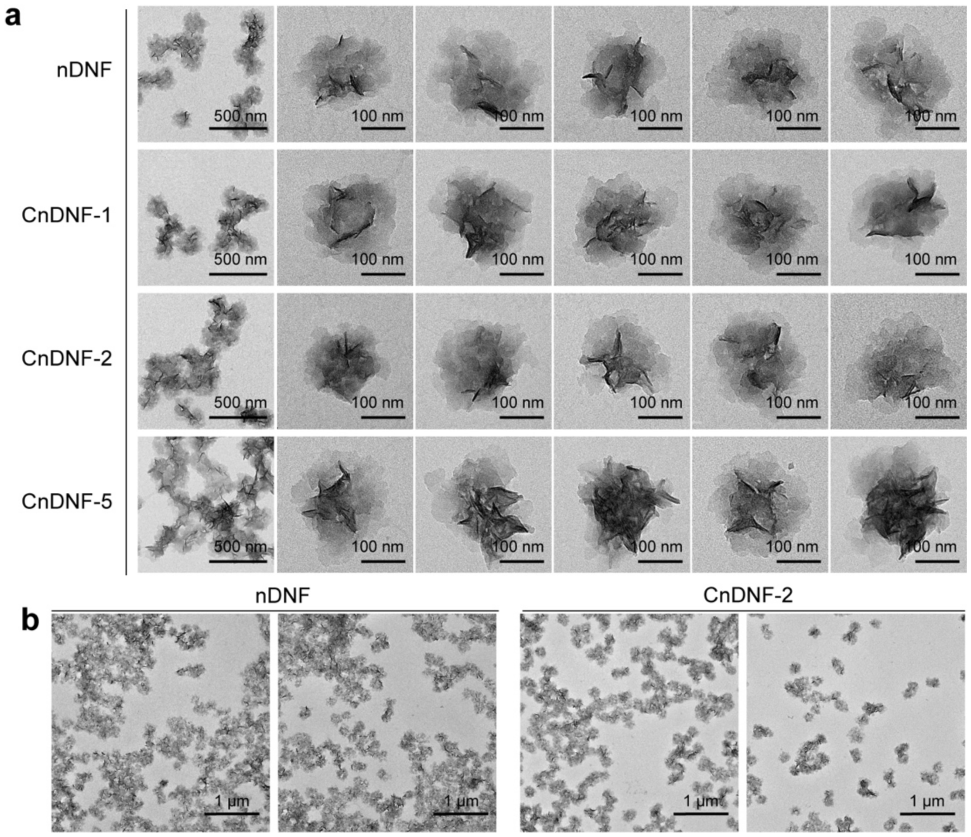

Figure S2. Characterization of CnDNF with TEM. (a) Representative TEM images of CnDNF fabricated with various Chol-DNA concentrations (1, 2 and 5 μM). HR-TEM images showed 200–300 nm sized particles with curved backbones and petal-like multilayers. (b) Representative TEM images of nDNF (left) and CnDNF-2 (right) at low magnification.

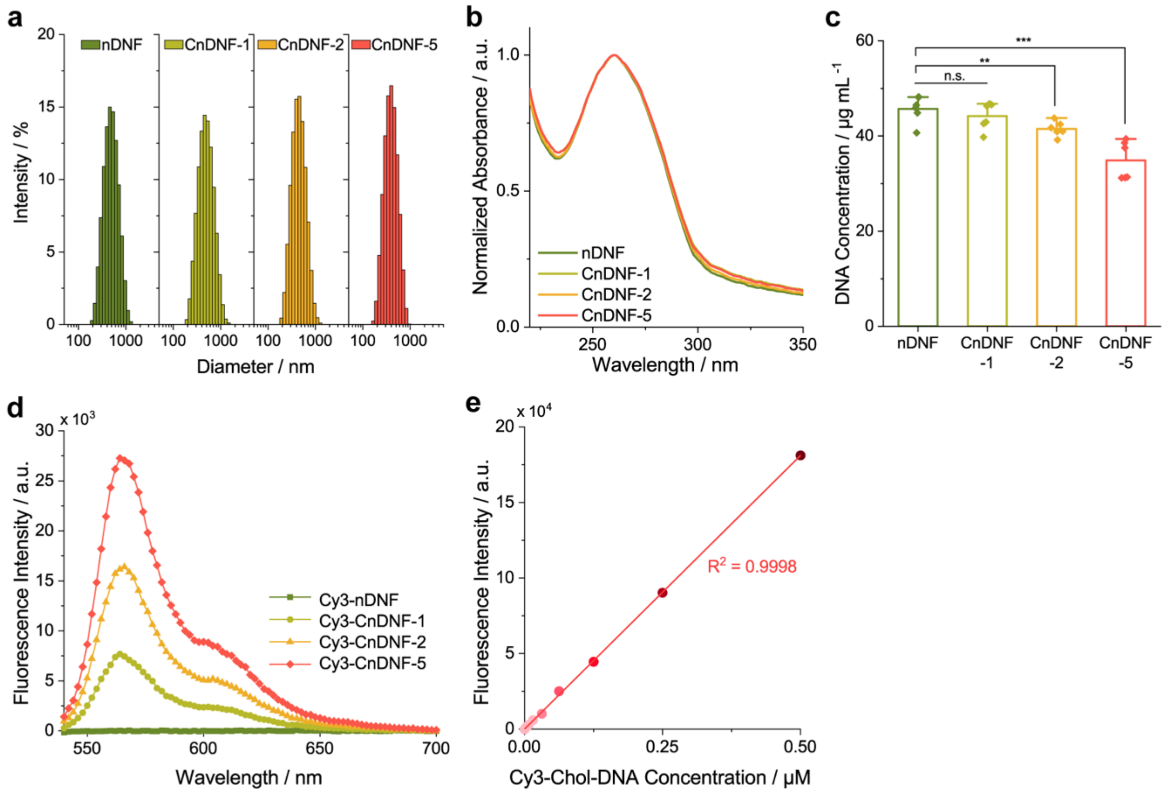

Figure S3. Characterization of CnDNF aptasensor. (a) Intensity particle size distribution of CnDNF measured by DLS. The slightly larger DLS hydrodynamic size (ca. 400 nm) compared to the sizes determined based on SEM and TEM (200–300 nm) presumably indicates the presence of a hydration layer of DNA flowers in aqueous media, where the amplified DNA strands serve as long polymeric hydrophilic organic molecules, which are likely highly swollen in a solvated state and exist throughout the construct. (b) UV absorbance of nDNF and CnDNF. The UV-vis absorption spectra of nDNF and CnDNF showed a single characteristic band at 260 nm, whereas marginally reduced absorbance ratios of 260 nm/280 nm were measured in CnDNF (ca. 1.39 in CnDNF-5) compared to that of nDNF (ca. 1.43). (c) Quantification of DNA content in nDNF and CnDNF. (d) Emission fluorescence spectra (excitation at 540 nm) of nDNF and CnDNF (200 ng μL^{-1} of DNA) fabricated with Cy3-Chol-DNA. (e) Calibration curve from the fluorescence intensity of Cy3-Chol-DNA solution used for the quantification of Cy3-Chol-DNA content. A straight line was fitted to the data with R-squared (R^2) indicated. Data represent mean ± s.d. or mean for (a, b) three repeated measurements, (c) six repeated assays across two independent experiments, (d) three independent experiments, or (e) two independent measurements. n.s. (not significant, $P > 0.05$), ***$p < 0.0001$, and **$p < 0.01$ based on one-way ANOVA and Tukey's honest significance test.

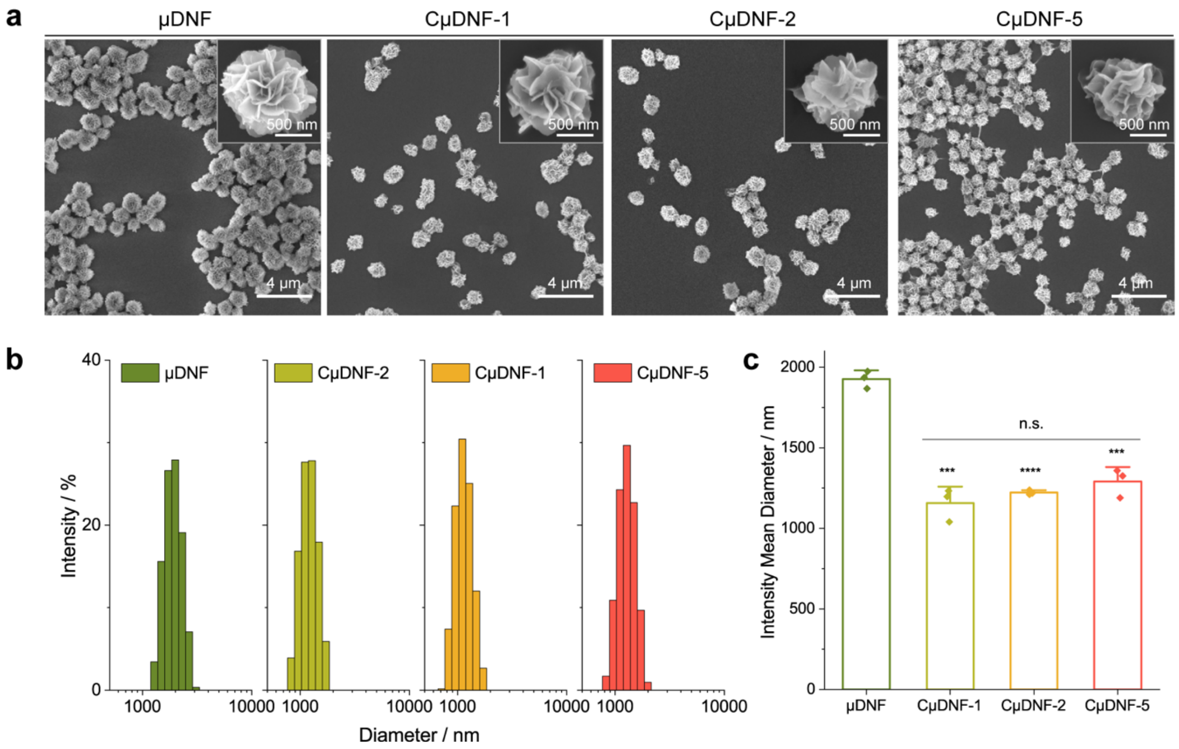

Figure S4. Effect of Chol-DNA addition in RCA mixture after 20 h of reaction. (a) Representative SEM images of DNA microflowers (μDNF) and cholesterol-decorated DNA microflowers (CμDNF) fabricated with various Chol-DNA concentrations (1, 2, and 5 μM) showing apparent size reducing effect by Chol-DNA: μDNF, CμDNF-1, CμDNF-2, and CμDNF-5. (b) Intensity particle size distribution of CμDNF determined by DLS (c) Intensity mean measured by DLS. Data represent mean or mean ± s.d. for three repeated measurements. n.s. (not significant, $P > 0.05$), ****$p < 0.0001$, and ***$p < 0.0001$ with respect to bare μDNF, based on one-way ANOVA and Tukey's honest significance test.

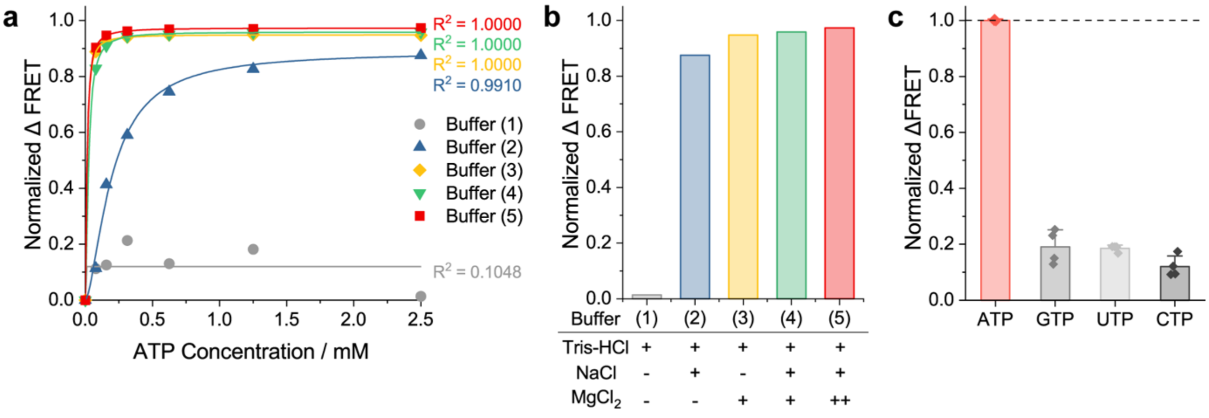

Figure S5. Ratiometric detection using fDNA aptasensors. (a) ATP dose-responsive ratiometric signal changes from fDNA in different reaction buffers. The buffer composition is: (1) 20 mM Tris-HCl, pH 7.5, (2) 20 mM Tris-HCl, 300 mM NaCl, pH 7.5, (3) 20 mM Tris-HCl, 5 mM $MgCl_2$, pH 7.5, (4) 20 mM Tris-HCl, 300 mM NaCl, 5 mM $MgCl_2$, pH 7.5, (5) 20 mM Tris-HCl, 300 mM NaCl, 10 mM $MgCl_2$, pH 7.5. Adapted from a previous report on structure-switching ATP aptasensor,[6] the fDNA was constructed with a ratio of DE:CP:AE = 1:2:3, where DE, CP, and AE refers to donor-labeled DNA element, capture probe, and acceptor-labeled DNA element, respectively. The fluorescence was measured after 1 h incubation at 37 °C. FRET ratio was estimated using the equation: FRET ratio = FI_{DA}/FI_D, where FI_{DA} and FI_D refer to acceptor emission maximum and donor emission maximum, respectively. The signal changes are defined as FRET ratio change ($\Delta FRET = FRET_0 - FRET_{ATP}$, where $FRET_{ATP}$ and $FRET_0$ are FRET ratio in the presence, and absence of a target, respectively), normalized with respect to $FRET_0$. Each profile was fitted with a sigmoidal curve (Hill fit) with the R-squared (R^2) indicated. (b) Bar graph showing signal changes in the presence of 2.5 mM ATP at various buffer conditions. (c) Specificity test of ratiometric signaling using 2.5 mM of ATP, uridine triphosphate (UTP), cytidine triphosphate (CTP), and guanosine triphosphate (GTP). FRET ratio change (ΔFRET) was normalized to that of ATP. Data represents mean ± s.d. for four independent measurements.

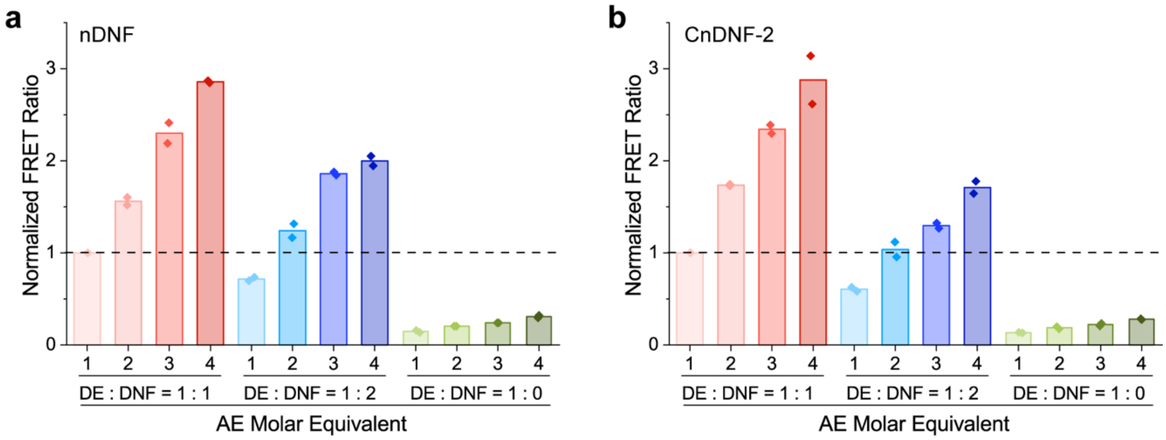

Figure S6. Effect of the molar ratio of each constituent of nDNF and CnDNF aptasensors. (a–b) FRET ratio upon hybridization of donor-labeled DNA element (DE) and acceptor-labeled DNA element (AE) into (a) nDNF or (b) CnDNF-2 constructs with different molar ratios. FRET ratio was estimated by following the equation: FRET ratio = FI_{DA}/FI_{D}, where FI_{DA} and FI_{D} refer to acceptor emission maximum and donor emission maximum, respectively. In order to clearly demonstrate the difference, the ratio was normalized with respect to the result from 1:1:1 molar ratio, exhibiting the highest FRET ratio when the molar ratio of DE:DNF:AE was 1:1:4. Fluorescence was measured at room temperature. Bars represent the mean of two independent measurements.

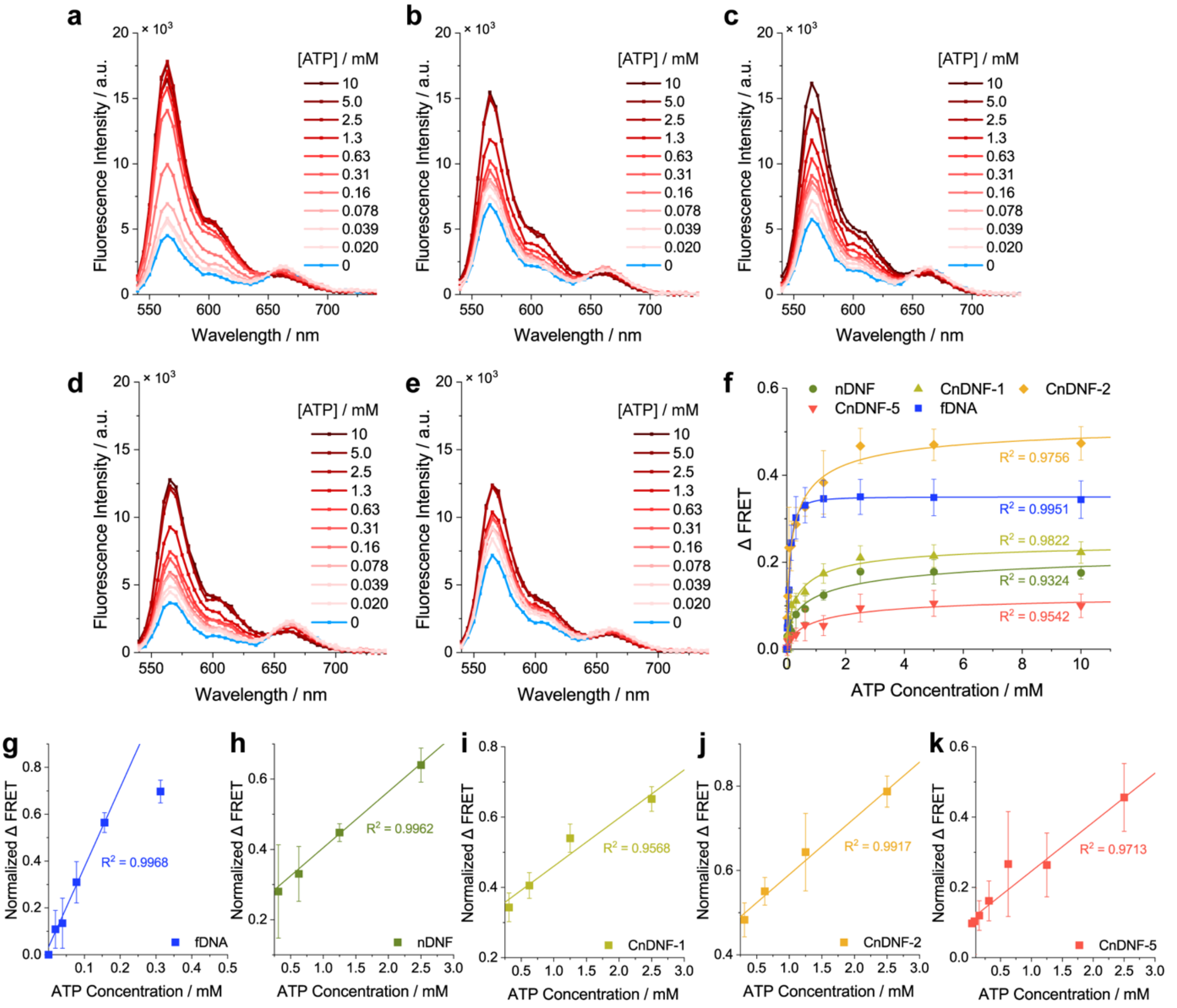

Figure S7. Ratiometric detection using CnDNF aptasensors. (a–e) Full emission fluorescence spectra of the aptasensors with varying concentrations of ATP: (a) fDNA, (b) nDNF, (c) CnDNF-1, (d) CnDNF-2, (e) CnDNF-5. (f) Dose-responsive ratiometric signal changes with varying concentrations of ATP. FRET ratio can be calculated from the results using the following equation: FRET ratio = FI_{DA} / FI_{D}, where FI_{DA} and FI_{D} refer to acceptor emission maximum and donor emission maximum, respectively. The signal changes are defined as non-normalized FRET ratio change ($\Delta FRET = FRET_0 - FRET_{ATP}$, where $FRET_{ATP}$ and $FRET_0$ are FRET ratio in the presence and absence of a target, respectively). (g–k) Linear detection range of the aptasensors in the dose-responsive curves (Figure 2i): (g) fDNA, (h) nDNF, (i) CnDNF-1, (j) CnDNF-2, (k) CnDNF-5. The signal changes are defined as FRET ratio change (ΔFRET) normalized with respect to $FRET_0$. Straight lines were fitted to the data with R-squared (R^2) indicated. All fluorescence measurements were carried out after 1 h of incubation at 37 °C and recorded at 37 °C. Data represent mean or mean ± s.d. for three independent experiments.

Fluorescence correlation spectroscopy (FCS)

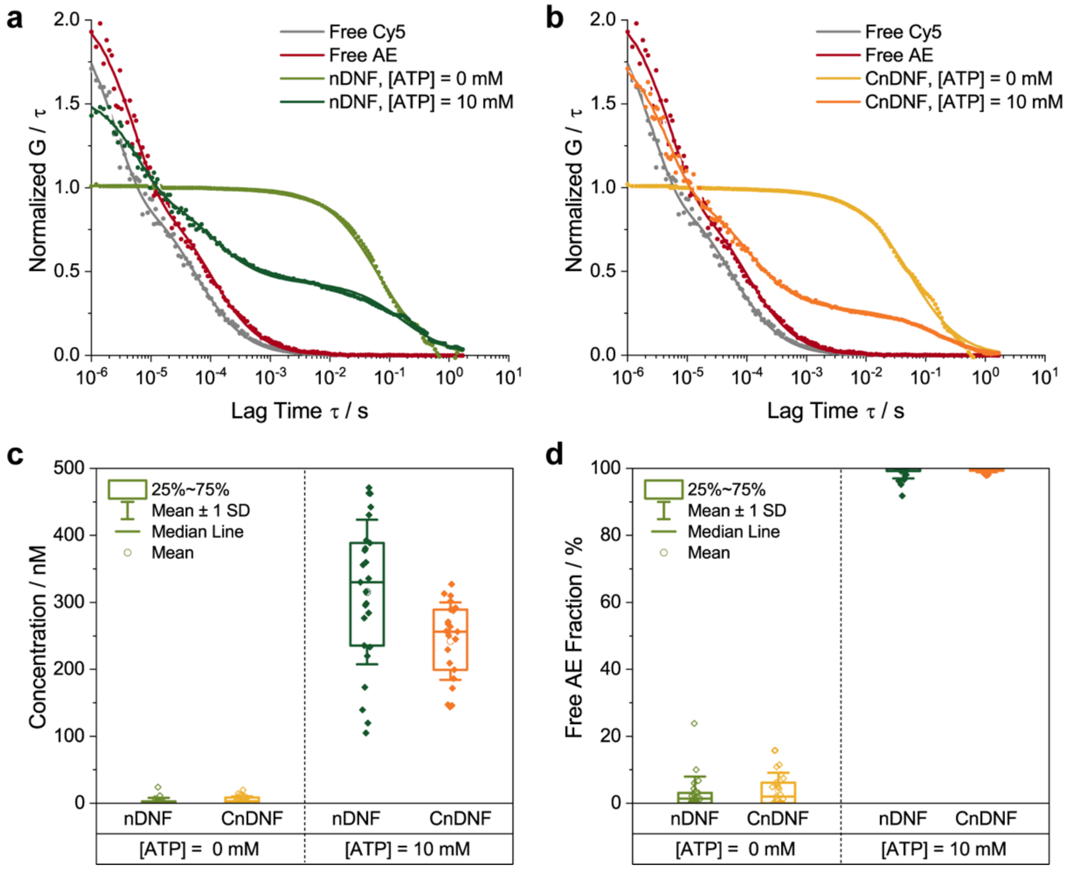

Figure S8. Validation of the target-triggered dissociation of acceptor-labeled DNA element (AE) from nDNF and CnDNF aptasensors using fluorescence correlation spectroscopy (FCS). (a–b) Normalized autocorrelation curves of (a) nDNF and (b) CnDNF-2 (CnDNF). FRET aptasensors from FCS measurements (Cy5 excited directly, 633 nm laser) in comparison with free Cy5 dyes and free AE in solution. The donor- and acceptor-DNA element (DE and AE)-hybridized aptasensors were incubated in the absence and in the presence of ATP (10 mM) at 37 ºC for 30 min prior to the measurements. Dots and solid lines refer to experimental and fits, respectively. (c) Concentration of diffusing species and (d) fraction of free AE liberated (see FCS in Experimental Section) from nDNF and CnDNF incubated without (left) and with ATP (10 mM) (right) for 30 min. Data represent mean or mean ± s.d. with median for 25 independent measurements.

To demonstrate the liberation of AE from DNF aptasensors in solution, we employed fluorescence correlation spectroscopy (FCS), which is a single-molecule detection technique that allows the identification of aptasensor-bound versus free AE diffusion. When exciting AE directly (633 nm laser), we observed a shift from nearly pure aptasensor diffusion (containing many AE strands per particle) to mostly free AE diffusion when ATP was present, which evidently indicates that the target-triggered liberation of AE occurred. This is further substantiated by the increase in the

concentration of diffusing species due to the liberation of a large amount of AE from nDNF and CnDNF. The two-component nature of the average autocorrelation curves (Figure S8a and S8b) in the presence of target is explained by the disproportional domination of FCS curves by slow diffusing species;[4] even a single AE left on any of the aptasensor particles will show up in the correlation curves. To obtain a more accurate fraction of the liberated AE, a correction was included, which combines the free fraction from the fits with the accompanying decrease in the numbers of AE per remaining aptasensor when compared to the data obtained in the absence of the target.[5] This yielded 99.0% and 99.6% of AE being liberated respectively from nDNF and CnDNF aptasensor in the presence of ATP (Figure S8c and S8d). Taken together, the FCS results clearly validate that the dissociation of AE from DNF constructs is triggered by target-binding to DNF aptasensor regardless of the cholesterol decoration.

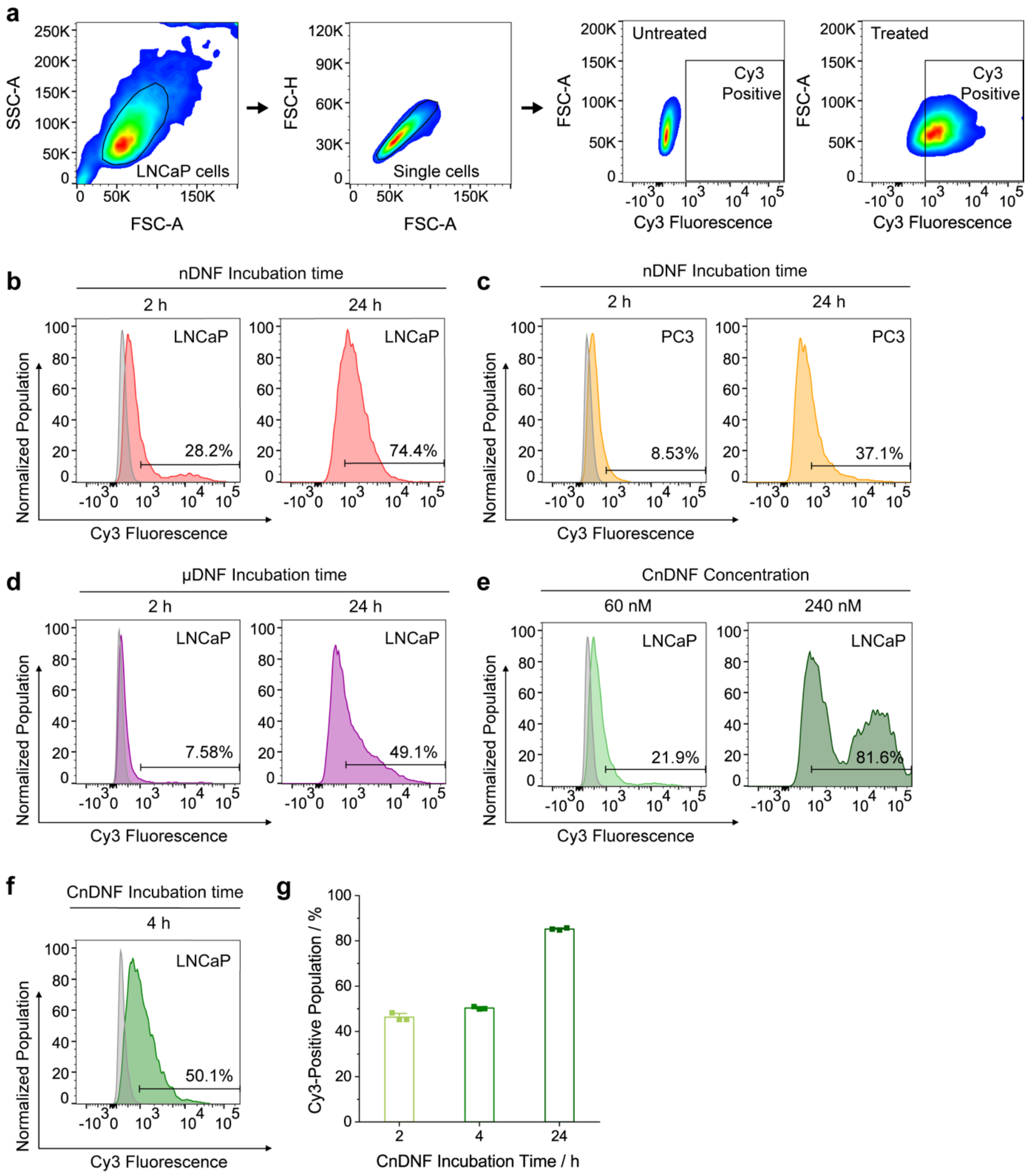

Figure S9. Flow cytometry investigation of cellular uptake of DNF. (a) Exemplary gating strategy to measure the Cy3-positive population, demonstrated by LNCaP after treatment with Cy3-labeled CnDNF (120 nM) for 24 h (SSC-A: side scatter area, FSC-A: forward scatter area, FSC-H: forward scatter height). (b–f) Representative histogram of cell population versus Cy3 fluorescence intensity measured by flow cytometry analysis after treatment with: (b–c) Cy3-labeled nDNF (120 nM) to (b) LNCaP and (c) PC3 cells for 24 h, (d) Cy3-labeled μDNF (120 nM) to LNCaP cells for 2 h, (e) Cy3-labeled CnDNF (60 and 240 nM) to LNCaP cells for 2 h, (f) Cy3-labeled CnDNF (120 nM) to LNCaP cells for 4 h. (g) Time-dependency of Cy3-positive population of LNCaP cells after treatment with Cy3-labeled CnDNF (120 nM) for 2h, 4 h, and 24h. Bars represent mean ± s.d. for three independent experiments. All particles (a–g) presented PSMA-binding aptamer (Apt). The sample treatment was carried out at 37 °C.

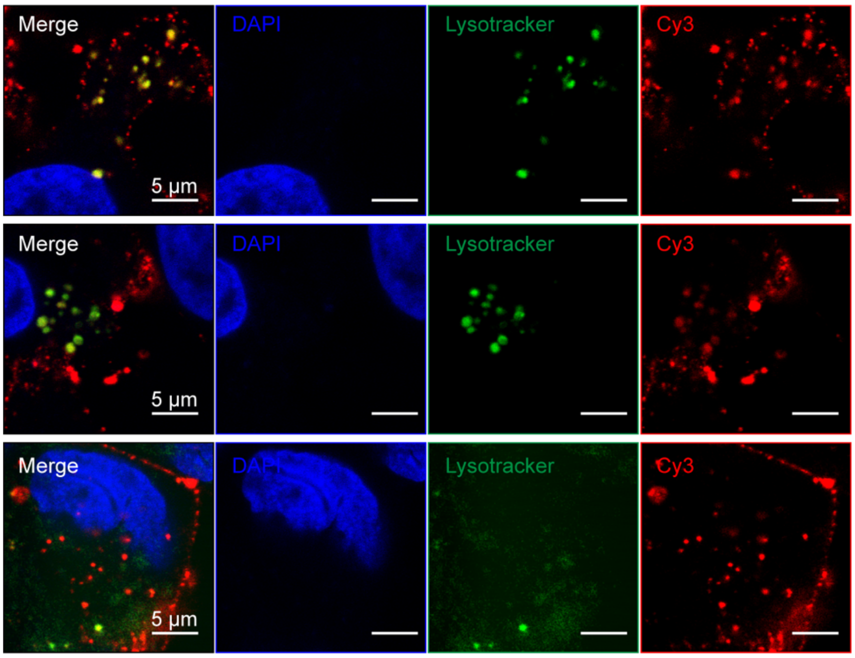

Figure S10. Intracellular colocalization of CnDNF with acidic cellular compartments. The LNCaP cells were treated with Cy3-labeled CnDNF (240 nM, red) at 37 °C for 2 h and subsequently stained with LysoTracker™ Green (green, lysosomes) at 37 °C for 2 h after removing the particles. The cells were also counterstained with DAPI (blue, nucleus).

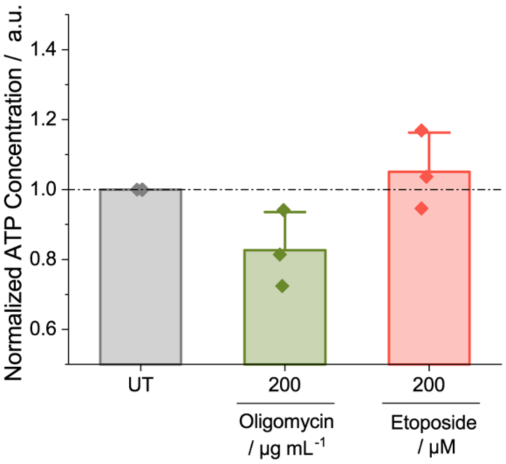

Figure S11. Luminescence-based examination of intercellular ATP levels in LNCaP cells using a commercially available kit. To alter the intracellular ATP level, LNCaP cells were pre-treated with oligomycin (200 μg mL^{-1}) or etoposide (200 μM) for 4 h at 37 °C, followed by performing an assay. The mechanism of the assay is based on the generation of light from the reaction of ATP in cell lysis upon the addition of a firefly's luciferase and luciferin. The data from drug-treated LNCaP were normalized with respect to untreated control and represent mean ± s.d. for three independent experiments (untreated control n = 6 per experiment). The smaller change at high concentration of etoposide is in agreement with the previous reports, indicating that the ensemble method typically underestimates the ATP level for apoptotic drugs as dead cells lack ATP.[7]

References:

[1] a) E. Kim, S. Agarwal, N. Kim, F. S. Hage, V. Leonardo, A. Gelmi, M. M. Stevens, *ACS Nano* **2019**, *13*, 2888; b) E. Kim, L. Zwi-Dantsis, N. Reznikov, C. S. Hansel, S. Agarwal, M. M. Stevens, *Adv. Mater.* **2017**, *29*, 1701086.

[2] P. Kapusta, Absolute Diffusion Coefficients: Compilation of Reference Data for FCS Calibration (Application Note, Rev. 1), PicoQuant GmbH, Germany, **2010**, https://www.picoquant.com/images/uploads/page/files/7353/appnote_diffusioncoefficients.pdf, accessed: December, 2020.

[3] P. Müller, P. Schwille, T. Weidemann, *Bioinformatics* **2014**, *30*, 2532.

[4] A. Tcherniak, C. Reznik, S. Link, C. F. Landes, *Anal. Chem.* **2009**, *81*, 746.

[5] L. Massi, A. Najer, R. Chapman, C. D. Spicer, V. Nele, J. Che, M. A. Booth, J. J. Doutch, M. M. Stevens, *J. Mater. Chem. B* **2020**, *8*, 8894.

[6] R. Nutiu, Y. Li, *J. Am. Chem. Soc.* **2003**, *125*, 4771.

[7] M. V. Zamaraeva, R. Z. Sabirov, E. Maeno, Y. Ando-Akatsuka, S. V. Bessonova, Y. Okada, *Cell Death Differ.* **2005**, *12*, 1390.
